# Genus-Wide Comparative Genomics of *Malassezia* Delineates Its Phylogeny, Physiology, and Niche Adaptation on Human Skin

**DOI:** 10.1371/journal.pgen.1005614

**Published:** 2015-11-05

**Authors:** Guangxi Wu, He Zhao, Chenhao Li, Menaka Priyadarsani Rajapakse, Wing Cheong Wong, Jun Xu, Charles W. Saunders, Nancy L. Reeder, Raymond A. Reilman, Annika Scheynius, Sheng Sun, Blake Robert Billmyre, Wenjun Li, Anna Floyd Averette, Piotr Mieczkowski, Joseph Heitman, Bart Theelen, Markus S. Schröder, Paola Florez De Sessions, Geraldine Butler, Sebastian Maurer-Stroh, Teun Boekhout, Niranjan Nagarajan, Thomas L. Dawson

**Affiliations:** 1 Computational and Systems Biology, Genome Institute of Singapore, A*STAR, Singapore; 2 Procter & Gamble Singapore Innovation Center, Singapore; 3 Bioinformatics Institute, A*STAR, Singapore; 4 Procter & Gamble Mason Business Center, Mason, Ohio, United States of America; 5 Translational Immunology Unit, Department of Medicine Solna, Karolinska Institutet and University Hospital, Stockholm, Sweden; 6 Duke University Medical Center, Durham, North Carolina, United States of America; 7 National Center for Biotechnology Information, Bethesda, Maryland, United States of America; 8 University of North Carolina at Chapel Hill, Chapel Hill, North Carolina, United States of America; 9 Fungal Biodiversity Centre, CBS-KNAW, Utrecht, The Netherlands; 10 University College Dublin, Dublin, Ireland; 11 School of Biological Sciences, Nanyang Technological University (NTU), Singapore; 12 Institute of Medical Biology, A*STAR, Singapore; Stanford University School of Medicine, UNITED STATES

## Abstract

*Malassezia* is a unique lipophilic genus in class Malasseziomycetes in Ustilaginomycotina, (Basidiomycota, fungi) that otherwise consists almost exclusively of plant pathogens. *Malassezia* are typically isolated from warm-blooded animals, are dominant members of the human skin mycobiome and are associated with common skin disorders. To characterize the genetic basis of the unique phenotypes of *Malassezia* spp., we sequenced the genomes of all 14 accepted species and used comparative genomics against a broad panel of fungal genomes to comprehensively identify distinct features that define the *Malassezia* gene repertoire: gene gain and loss; selection signatures; and lineage-specific gene family expansions. Our analysis revealed key gene gain events (64) with a single gene conserved across all *Malassezia* but absent in all other sequenced Basidiomycota. These likely horizontally transferred genes provide intriguing gain-of-function events and prime candidates to explain the emergence of *Malassezia*. A larger set of genes (741) were lost, with enrichment for glycosyl hydrolases and carbohydrate metabolism, concordant with adaptation to skin’s carbohydrate-deficient environment. Gene family analysis revealed extensive turnover and underlined the importance of secretory lipases, phospholipases, aspartyl proteases, and other peptidases. Combining genomic analysis with a re-evaluation of culture characteristics, we establish the likely lipid-dependence of all *Malassezia*. Our phylogenetic analysis sheds new light on the relationship between *Malassezia* and other members of Ustilaginomycotina, as well as phylogenetic lineages within the genus. Overall, our study provides a unique genomic resource for understanding *Malassezia* niche-specificity and potential virulence, as well as their abundance and distribution in the environment and on human skin.

## Author Summary

*Malassezia* are the dominant eukaryotic residents of human skin and are associated with the most common skin disorders, including dandruff, atopic dermatitis, eczema, and others. Despite significant effort, the role of *Malassezia* in skin disease and homeostasis remains unclear. *Malassezia* are also unique among fungi by requiring lipids for growth, but the breadth and genetic basis of their lipophilic lifestyle has not been comprehensively studied. Here we report the complete genomes of all 14 *Malassezia* species (including multiple strains of the most common species found on humans) and systematically identify features that define the genus and its sub-lineages, including horizontally transferred genes likely to represent key gain-of-function events and which may have enabled evolution of the genus from plant to animal inhabitants. Genus wide expansion of lipid hydrolases and loss of carbohydrate metabolism genes underscore the entire genus’ gradual evolution to lipid-dependency, which was confirmed even in the previously thought to be lipophilic *M*. *pachydermatis*, via genomics with experimental confirmation. Finally, these reference genomes will serve as a valuable resource for future metagenomic investigations into the role of *Malassezia* species in normal healthy skin and diseases.

## Introduction

Over 100 years ago *Malassezia* was recognized as an inhabitant of human skin and implicated in a common skin disorder i.e. seborrheic dermatitis [1]. Since then, *Malassezia* has been found on the skin of all tested warm blooded animals [2,3], including dogs, horses, pigs, goats, cats and lambs [4–8], and associated with other common skin disorders including dandruff [9], atopic eczema/dermatitis, pityriasis versicolor, seborrheic dermatitis, and in systemic disease [10]. Recent investigations of the skin microbiome using culture-free approaches have highlighted the overwhelming dominance of *Malassezia* among eukaryotes on all human surface body sites, with only the exception of three foot sites [11,12]. Other studies have suggested that they are abundant in body sites beyond skin, including the human oral microbiome [13], but a systematic characterization of *Malassezia* species and their functional repertoires represented in metagenomic datasets has been hampered by the lack of reference genomes (only 2 out of 14 known species have reference genomes i.e. *M*. *globosa* [2] and *M*. *sympodialis* [14]). In addition, several reports have suggested that *Malassezia*-like organisms are found in a wide range of environmental habitats, from deep sea sediments, hydrothermal vents and arctic soils, to marine sponges, stony corals, eels, lobster larvae, and nematodes [15]. These studies have relied on high-identity DNA sequence matches to short amplified barcode regions, but concerns about amplification bias or laboratory contamination raise doubts about the results and the lack of a comprehensive genus-wide genomic resource for known species has made it challenging to investigate this question further.

*Malassezia* belong to the class Malasseziomycetes in the subphylum of Ustilaginomycotina, (phylum of Basidiomycota, Kingdom of Fungi) [16], which are otherwise comprised exclusively of more than 1,500 species of plant pathogens [17]. Other known fungal residents on human skin, such as *Candida albicans* and the dermatophytes are in distant branches of the fungal tree of life and are likely to have evolved independently to adapt to life on animal skin [18,19]. The genetic basis of the unique lipophilic nature of *Malassezia* and its adaptation to animal skin (putatively starting from an ancestral state as a plant or soil resident) is thus an intriguing and open question. Answers to this question could also serve as the basis for developing new anti-fungals and therapeutics for associated skin disorders. Analysis of the two existing *Malassezia* genomes [2,14] highlights that their small genomes (among the smallest for free living organisms in the fungal kingdom) likely contain only the minimal complement of information necessary for existence in their specific ecological niche. In this context, the expansion of several gene families as noted before (e.g. lipases, phospholipases, and aspartyl proteases) may point to their functional importance [2,14]. However, it has not been clear if these observations are indeed genus-wide features. In addition, the limited availability of reference genomes has precluded the systematic characterization of genomic features unique to *Malassezia* (such as gene gain or loss, horizontal gene transfers, linkage between mating type loci, and regions undergoing positive or negative selection) that could serve as the basis of understanding its unique physiology and niche adaptation.

To address this limitation, we sequenced and assembled high-quality, annotated genomes of all known *Malassezia* species and multiple strains of the species most common on humans (including a re-annotation of existing references), representing a 7-fold increase in available reference genomes (from 2 to 14), and providing a comprehensive genomic resource for the investigation of *Malassezia* biology and its ecological distribution (24 *Malassezia* strains in total). Showcasing this, we established the abundance and surprising diversity of *Malassezia* species on various human skin sites and their scarcity in other environments. We then used comparative genomic analysis to systematically compare *Malassezia* genomes with a broad panel of fungal genomes to reveal genomic features unique to *Malassezia*, including hundreds of gene gain and loss events, gene family expansions, and positive selection events. Our analysis revealed several hallmarks of *Malassezia* genomes, including key horizontally transferred genes (a few of bacterial origin) that we characterized functionally and which may be prime candidates to explain the emergence of host and niche-adaptation in *Malassezia*. A larger set of genes (>700) were found to be lost in all *Malassezia* compared to other Basidiomycota, with an enrichment for glycosyl hydrolases and genes involved in carbohydrate metabolism, concordant with adaptation to a carbohydrate-deficient environment. Combining genomic analysis with an experimental re-evaluation of culture characteristics, we revert previous assumptions and established the likely lipid dependency of all *Malassezia* species. Finally, our analysis of lineage-specific gene family expansions revealed extensive turnover in the gene repertoire of *Malassezia* and underlined the importance of secretory lipases, phospholipases, aspartyl proteases and other peptidases in the experimentally observed lipid specificity of this genus.

## Results

### Establishing a comprehensive genomic resource for the *Malassezia* genus

Genome sequences for all 14 known *Malassezia* species, including multiple strains of the more widely studies species (24 in total) were obtained by high-throughput sequencing and *de novo* assembly (Table 1; see Methods). The high coverage data (median coverage of 322X) was systematically assembled with an assembly pipeline incorporating parameter optimization, contig construction, scaffolding and gap closure steps to produce assemblies with a median N50 of 54 kbp and a maximum N50 of 1.4 Mbp (Table 1). In particular, we noted that the N50s of the four new *M*. *globosa* assemblies were comparable to that of a gold-standard reference *M*. *globosa* genome [2] obtained previously using Sanger sequencing with significant directed finishing **(** Table 1). Assembly sizes typically varied from 7.2 Mbp (for *M*. *restricta*) to 9.0 Mbp (for *M*. *globosa*) as expected but we noted that 4 out of the 6 *M*. *furfur* assemblies were twice this size, suggesting that they might have undergone whole genome duplication or hybridization events (see S1 Text and S1 Fig for further details).

**Table 1:** Assembly and annotation statistics for *Malassezia* genomes in this study. Note the statistics for the previously reported *M*. *globosa* [2] and *M*. *sympodialis* [14] assemblies are provided for reference.

Species	Strain	Naming source*	No. of reads (in millions)	Coverage	Assembly size (Mbp)	N50 (kbp)	No. of genes
*M*. *caprae*	10434	CBS	18	479X	7.6	110	3925
*M*. *cuniculi*	11721	CBS	11	298X	7.5	522	4112
*M*. *dermatis*	9169	CBS	13	357X	7.5	189	3890
*M*. *equina*	9969	CBS	9	244X	7.7	372	4109
*M*. *furfur*	1878	CBS	12	176X	13.5	15	9827
	4172	CBS	19	271X	14.0	16	10232
	7019	CBS	14	207X	13.4	16	9612
	7710	CBS	14	189X	14.8	15	10980
	JPLK23	TB	15	389X	7.6	15	5660
	7982	CBS	19	500X	7.7	21	5357
*M*. *globosa*	7990	CBS	7	168X	8.9	415	4577
	7966	CBS	20	460X	8.9	724	4207
	7874	CBS	9	191X	8.9	398	4598
	reference 7966	CBS	NA	NA	9.0	654	4223
*M*. *japonica*	9431	CBS	15	345X	8.3	66	4715
*M*. *nana*	9557	CBS	16	433X	7.6	492	4242
*M*. *obtusa*	7876	CBS	18	455X	7.7	23	5028
*M*. *pachydermatis*	1879	CBS	19	463X	8.2	957	4328
*M*. *restricta*	7877	CBS	11	288X	7.2	403	4001
	8742	CBS	28	767X	7.3	667	4122
*M*. *slooffiae*	7956	CBS	11	262X	8.3	16	5617
*M*. *sympodialis*	42132	ATCC	10	280X	7.5	54	4390
	44340	ATCC	15	400X	7.5	60	4295
	96806	ATCC	14	360X	7.4	45	4541
	reference 42132	ATCC	NA	NA	7.7	513	4017
*M*. *yamatoenis*	9725	CBS	10	235X	8.1	1448	4361

*CBS: CBS-KNAW Fungal Biodiversity Centre, http://www.cbs.knaw.nl/;

TB: Teun Boekhout;

ATCC: The Global Bioresource Center, www.atcc.org.

**Fig 1:**
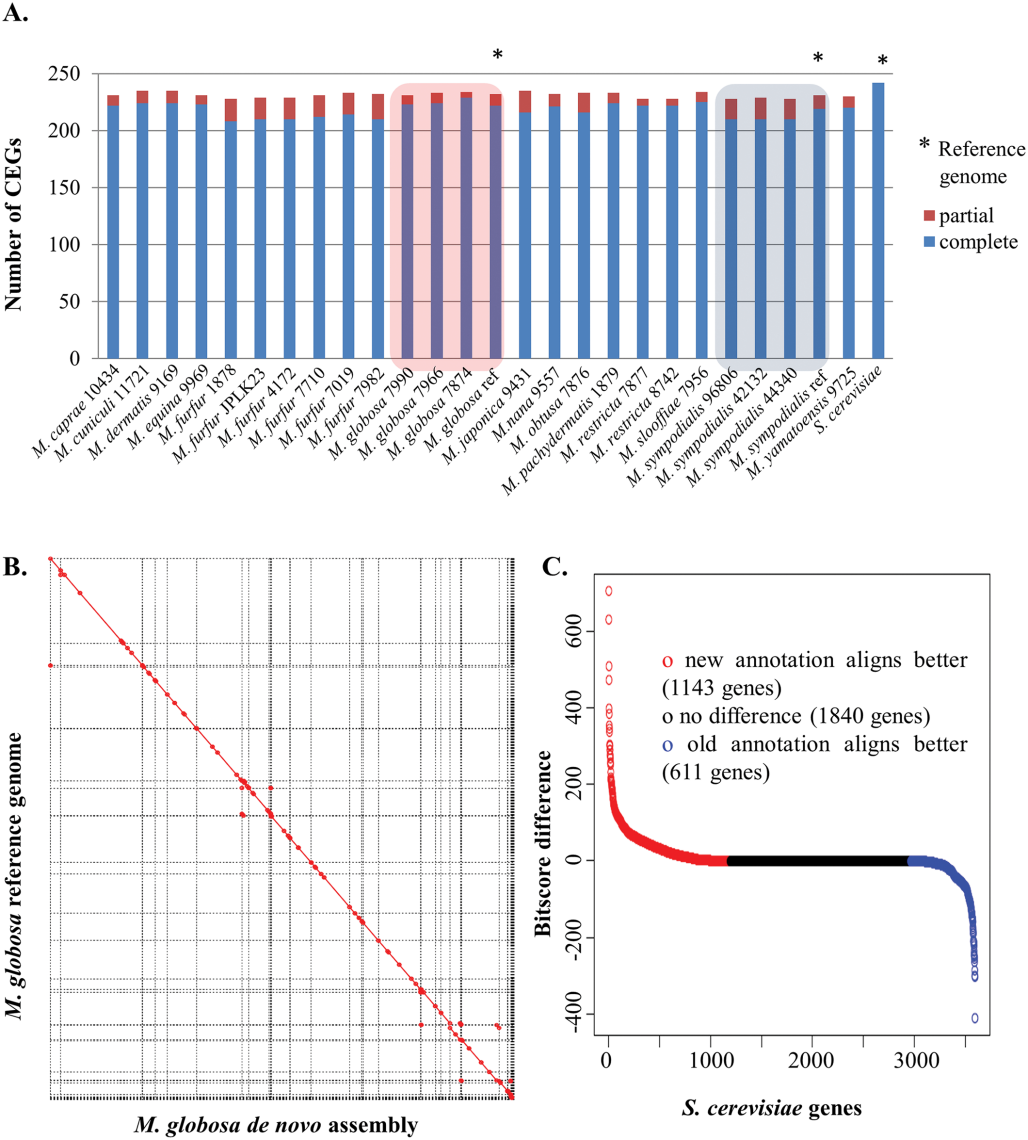
Correctness and completeness of *Malassezia* assembly and annotation. A) Assembly completeness in terms of partial and complete core eukaryotic genes that can be detected in each genome. As shown here, the assemblies from this study are comparable to published references for *M*. *globosa* and *M*. *sympodialis* and are very similar to the gold-standard *S*. *cerevisiae* genome. B) Whole-genome alignment of the assembly of *M*. *globosa* 7966 in this study as compared to the published reference, highlighting the robust assembly and the lack of clear misassemblies. C) Comparison of an annotation of *M*. *globosa* 7966 in this study with the reference annotation, using alignments to *S*. *cerevisiae* as a gold-standard. Y-axis indicates the BLAST bitscore difference between the top matches from the new and old annotations to the same *S*. *cerevisiae* protein. X-axis indicates the number of *S*. *cerevisiae* proteins. Red circles indicate *S*. *cerevisiae* proteins with a better match to the new annotation. Blue circles indicate *S*. *cerevisiae* proteins with a better match to the reference annotation.

To assess the completeness of our assemblies we evaluated them using matches to a well-established set of core eukaryotic genes (CEGs) [20]. As can be seen in Fig 1A, our *de novo* assemblies are comparable to the reference genomes of *M*. *globosa* and *M*. *sympodialis* [2,14] in terms of the number of complete and partial CEGs identified. In addition, comparison to the gold standard *Saccharomyces cerevisiae* genome suggests that our assemblies are more than 95% complete (Fig 1A). We assessed the correctness of our assemblies by comparing them to the published reference genomes of the same strains (*M*. *globosa* 7966 and *M*. *sympodialis* 42132). Overall, we found that our assemblies agreed very well with that of the reference genomes (Fig 1B, S1 Table), showing a high degree of colinearity (<5 breakpoints per 100 kbp in our assemblies) and identity (>99.92%) as expected from the comparison of two high-quality assemblies of the same strain (Fig 1B, S1 Table). Assuming that the reference genome is correct, we noted that the observed differences in our assembly affected <0.5% of all genes in *M*. *globosa*, indicating that our assembly is of particularly high quality in genic regions.

To systematically annotate the protein-coding complement of the genomes, we used an iterative and automated pipeline that combines transcriptome data (where available), *ab initio* predictions, and protein evidence from related species (see Methods). We evaluated results from this pipeline by comparison to the manually curated annotations for the *M*. *globosa* 7966 reference genome and using the *S*. *cerevisiae* annotations as gold standard. As shown in Fig 1C, as a whole, annotations from our pipeline match the *S*. *cerevisiae* proteome better (>1,100 *S*. *cerevisiae* proteins are better aligned to the new annotation versus ~600 proteins for the reference annotation) indicating that we have a comparable or better annotation. In addition, the new annotation has more matches to known domain families than the original annotation (unique PFam domains and total PFam domains, pfam.xfam.org/ Table 2) as well as improved identification of intron-exon boundaries, highlighting the value of the iterative approach employed here (the utility of transcriptome data is highlighted in S2 Table and the lack of alternative isoforms is noted in Methods **)**. As observed before, we found that *Malassezia* species code for a compact proteome of ~4,000 genes with the exception of *M*. *slooffiae* and *M*. *furfur* (after excluding those with doubled genome sizes) which appear to have a somewhat larger set of genes (Table 1). It is of note that the lower N50 of the *M*. *furfur* and *M*. *slooffiae* assemblies may cause a spurious increase in gene count due to coding regions being split.

**Table 2:** Comparison of annotation quality for the iterative annotation pipeline in this study with a reference annotation. Results shown are for the *M*. *globosa* genome.

		Reference	Iterative annotation
**General Statistics**	**# of genes**	4286	4271
	**Avg. length of genes**	1484	1613
	**# of exons**	6377	8214
	**# nucleotides in exons**	6.2 Mbp	6.6 Mbp
	**Exons per gene**	1.5	1.92
	**Avg. length of exons**	975	801
**Proteome Completeness**	**PFam domains**	7113	7327
	**Unique PFam domains**	2643	2755
**Intron-Exon Junctions**	**# of introns**	2092	3943
	**Introns per gene**	0.5	0.92
	**Avg. length of introns**	76	79
	**Junctions**	2091	3940
	**Supported junctions**	1785 (85%)	2995 (76%)

**Fig 2:**
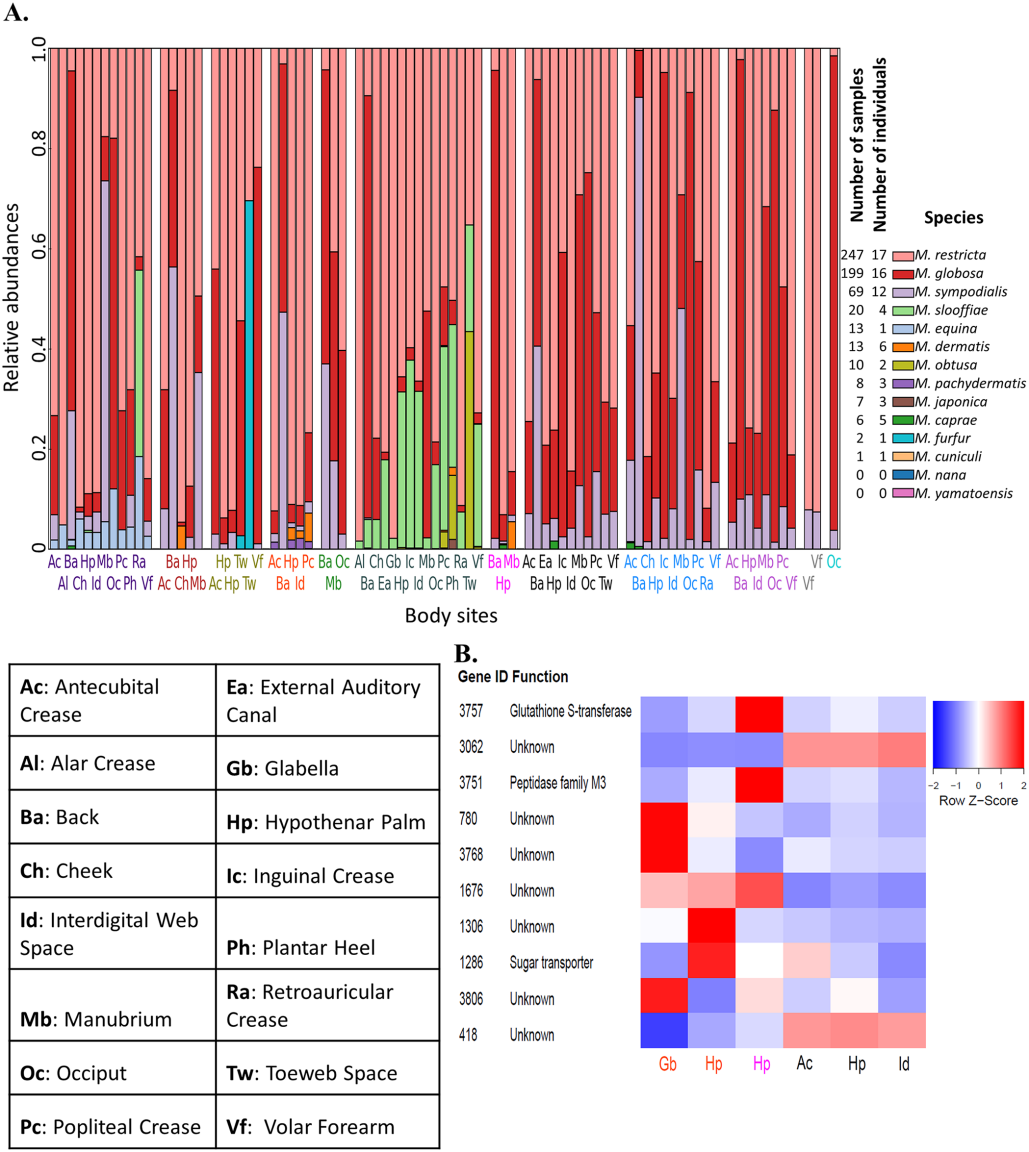
Characterizing the diversity of *Malassezia* in skin samples. A) The relative abundance of various *Malassezia* species (y-axis) in skin samples from different body sites (labels on the x-axis) and individuals (separated by white columns) is depicted. Samples where >99% of reads came from *M*. *globosa* and *M restricta* are not shown here. The numbers of samples and the numbers of individuals in which each species was found is indicated in the legend on the right. B) Z-score transformed normalized read counts for the top 10 copy number variable genes in *M*. *restricta* 7877 (measured in terms of coefficient of variation of normalized counts) across six skin samples.

To showcase the utility of this genomic resource, we studied the distribution and diversity of *Malassezia* species in the environment and in human microbiomes by extensive reanalysis of publicly available metagenomic datasets. We first used *in silico* benchmarks to confirm our analysis pipeline is highly sensitive and specific in identifying *Malassezia* species from short, shotgun metagenomic reads (S2 Fig). We then applied this approach to a wide spectrum of environmental metagenomic datasets including ocean (www.microb3.eu/osd), marine sediments [21], soil [22], and rhizosphere samples [23]. Despite recent reports of *Malassezia*-like organisms being widely distributed in the environment [15], we were unable to detect evidence for this, suggesting they are present in abundances below our detection limit or are sufficiently diverged from known *Malassezia* species to elude our detection based on known genomes. Similarly, we re-analyzed oral microbiome samples from six different oral sites (five samples from each site) and found no evidence for the presence of *Malassezia*, in contrast to a recent report [13]. These results are consistent with either contamination artefacts or *Malassezia* being presented at lower abundance in the oral mycobiome and thus being detectable only using more sensitive 18S rRNA sequencing approaches which utilize an amplification step [13]. In contrast, analysis of metagenomic datasets from different sites on healthy human skin [12] readily revealed the abundance and diversity of *Malassezia* (detected in 247 out of 280 samples analyzed, from 18 sites on 15 adults and two children; Fig 2A). In general, our analysis reconfirmed that *M*. *globosa* and *M*. *restricta* are the two most abundant species on human skin, found in 199 samples on 16 individuals and 247 samples on 17 individuals, respectively. *M*. *sympodialis* is a distant third, detectable in 69 samples on 12 individuals, though it is the most abundant species in several samples (Fig 2A). In addition, nine other species were also found either less frequently or in lower abundance. For example, *M*. *slooffiae*, which has previously not been detected on human skin via ribosomal RNA sequencing [11], was found in high abundance in several samples, mostly from one individual (Fig 2A). It is also accompanied in three samples by *M*. *obtusa* (in one individual) and apparently excluding *M*. *sympodialis* (in two individuals) (Fig 2A).

We further probed the relative abundance of various genes from the *Malassezia* pan-genome in skin metagenomic samples [12] to identify those that are highly variable, likely reflecting strain-level variations in the commensal population [24]. As *M*. *restricta* is the most abundant species on human skin, we were readily able to find samples with sufficient read coverage of the genome (>5X) for robust analysis (see Methods). Genome-wide we found significant copy number variations in >100 genes across 6 skin samples and 4 body sites (Fig 2B, S3 Table), though analysis of more samples is likely to reveal even more variable genes. Our proof-of-concept analysis revealed several highly variable genes including genes of unknown function, a glutathione S-transferase (known to be involved in detoxification of xenobiotic substrates), a peptidase and a sugar transporter (Fig 2B). As changes in carbohydrate and lipid metabolism are key features of *Malassezia* genomes (see Results below), this analysis suggests our reference genomes will serve as an important resource for characterizing strain variations contributing to different phenotypes on human skin.

### Identifying genetic features that define the *Malassezia* genus

**Fig 3:**
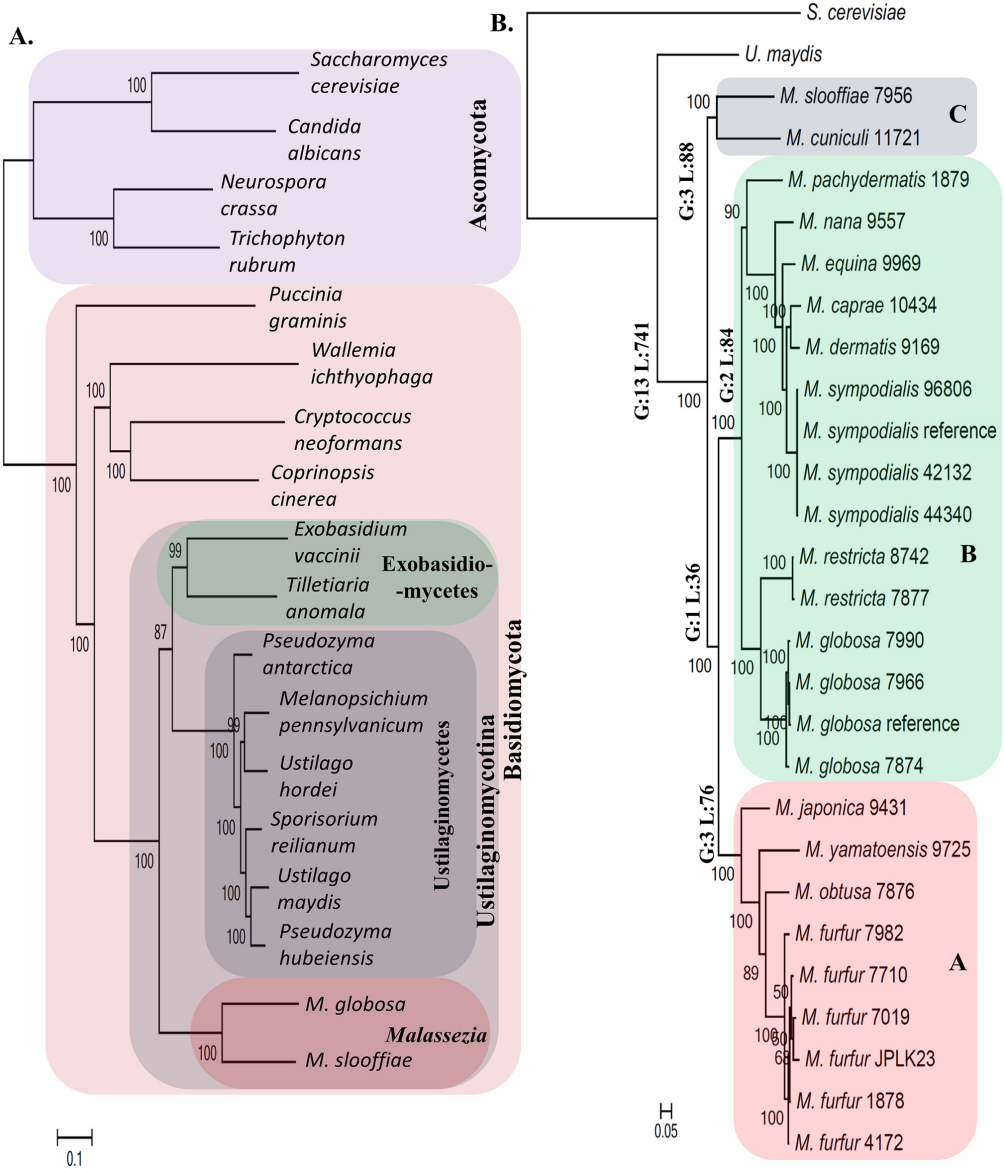
Phylogenetic relationships and lineage specific events in the *Malassezia* genus. A) The relationship of the *Malassezia* genus with respect to other fungi with sequenced genomes. *Malassezia* seem to form a distinct group in the subphylum of Ustilaginomycotina contrary to earlier reports. B) An expanded phylogeny of *Malassezia* that includes all known species in the genus. Major lineages of the genus are annotated with the number of lineage specific events that were identified in this study (G: gene family gain; L: gene family loss based on PFam analysis; see S3 Table for details). Horizontal numbers on each branch are bootstrap values.

Leveraging the comprehensiveness of our genomic resource for *Malassezia*, we set out to compare it against a broad panel of 16 fungal genomes (including all sequenced species in Ustilaginomycotina, a few other Basidiomycetes and several Ascomycetes as outgroups). Using a genome-wide multi-gene approach we first established a robust phylogenetic view of *Malassezia’s* relationship with other fungi and each other (Fig 3A and 3B; see Methods). In contrast to earlier reports placing *Malassezia* among the Exobasidiomycetes [17] or Ustilaginomycetes [25], our analysis suggests that it may be an isolated group (namely the class Malasseziomycetes) in the subphylum of Ustilaginomycotina, in agreement with Wang et al [16]. However, Wang et al placed Ustilaginomycetes close to *Malassezia*, and placed Exobasidiomycetes as the basal group [16]; our tree placed *Malassezia* as the basal group, indicating early divergence from its plant-pathogenic relatives. Within *Malassezia*, our phylogeny supports three main clusters (Fig 3B): Cluster A consists of fungemia-causing species *M*. *furfur* [26] and three other species (*M*. *japonica*, *M*. *obtusa*, and *M*. *yamatoensis*), rarely found on healthy human skin (Fig 2A); Cluster B includes a sub-cluster of the most common human skin residents *M*. *globosa* and *M*. *restricta* [11], the slightly less common *M*. *sympodialis* [14]) as well as related species in another sub-cluster; Cluster C consists of two outliers, *M*. *cuniculi* and *M*. *slooffiae* (Fig 3B), both of which are rare on human skin (Fig 2A). Notably, while broadly in agreement, our phylogeny disagrees with the placement of several species compared to a four-gene tree [27] and an earlier AFLP based tree [28], though its concordance with the mitochondrial phylogeny as well as alternative approaches to reconstruct phylogeny (S3 Fig) suggest that it is likely to be more reliable. Note that, as expected, our phylogeny also confirms the definition and molecular distinctness of various *Malassezia* species as well as the entire genus.

We then used comparative genomics to reveal genomic elements unique to *Malassezia*, identifying a small set of 13 functional domains (PFam families [29]) to be *Malassezia*-specific, compared to a much larger set of 741 domains likely lost in the common ancestor to all *Malassezia* (Fig 3B, S3 Table; see Methods), in addition to gene family expansions and signatures of selection (S3 Table). The set of *Malassezia*-specific genes contains mainly genes of unknown function and is not enriched for a specific functional category (S3 Table). On the other hand, the set of genes lost in all *Malassezia* varies widely in function, from genes encoding enzymes to transcriptional regulators to known accessory genes (S3 Table). However, we did detect significant enrichment for two lost functional categories, specifically, enzymes involved in carbohydrate metabolic process (q-value < 4.5×10^−4^) and in hydrolysis activity (hydrolyzing O-glycosyl compounds; q-value < 4.5×10^−4^), as expected for a genus of skin-adapted fungi that use lipids as their main carbon source. In addition, we also noted that the gene encoding the fatty acid synthase (FAS) was missing in all *Malassezia*, indicating that the genus is lipid-dependent and not just lipophilic as suggested earlier [30]. The idea that a subset of *Malassezia* is not lipid-dependent is based on the observation that some *M*. *pachydermatis* isolates can grow in media (Sabouraud-dextrose agar) without added lipids, though it does require fatty acids to grow in simple defined media [31]. We experimentally re-investigated the contents of Sabouraud-dextrose agar media and noted that the added peptone contains 0.6% lipid, with 6 μg of palmitic acid per gram of peptone and lesser amounts of other fatty acids. Furthermore, in 2 X YNB defined media, *M*. *pachydermatis* strains (1879 and 7550) were able to grow only in the presence of added lipids confirming the unique lipid-dependent nature of all *Malassezia* species **(** S2 Text).

At the structural level we confirmed linkage between the two mating loci (*MAT*) in three *Malassezia* species (belonging to clusters A and B, S4 Fig; i.e. likely a pseudo-bipolar configuration), a feature that is hypothesized to contribute to pathogenesis [32], but is unique to *Malassezia* among Basidiomycetes (S3 Text, S4 Fig and S4 Table). We also noted a loss of the RNAi pathway and a concomitant reduction in transposon element density in all *Malassezia* genomes (S4 Text). Finally our selection analysis revealed a diverse set of noteworthy genes undergoing positive selection (S5 Text and S3 Table), with the strongest signal being observed in a protein (with match to the PFam domain PF12481) known to be induced by aluminum, a common component of deodorant, shaving cream and gel [33].

### Acquisition and function of horizontally transferred genes in the *Malassezia* genus

The *Malassezia*-specific gene families identified using known domain families (PFam) contain many interesting candidates for horizontally transferred genes (HTGs). We also used a clustering based approach to expand this analysis to gene families with or without PFam domains, obtaining an additional set of 44 *Malassezia*-specific gene clusters, most of which have unknown function (S3 Table). Finally, we used two additional approaches based on similarity searches and phylogenetic analysis to catalog genes with more subtle evidence of horizontal transfer from bacteria into *Malassezia* [34,35] to identify 6 additional genes, many of which appear to be associated to oxidative stress response (including two oxidoreductases and one catalase; S3 Table and S6 Text).

**Fig 4:**
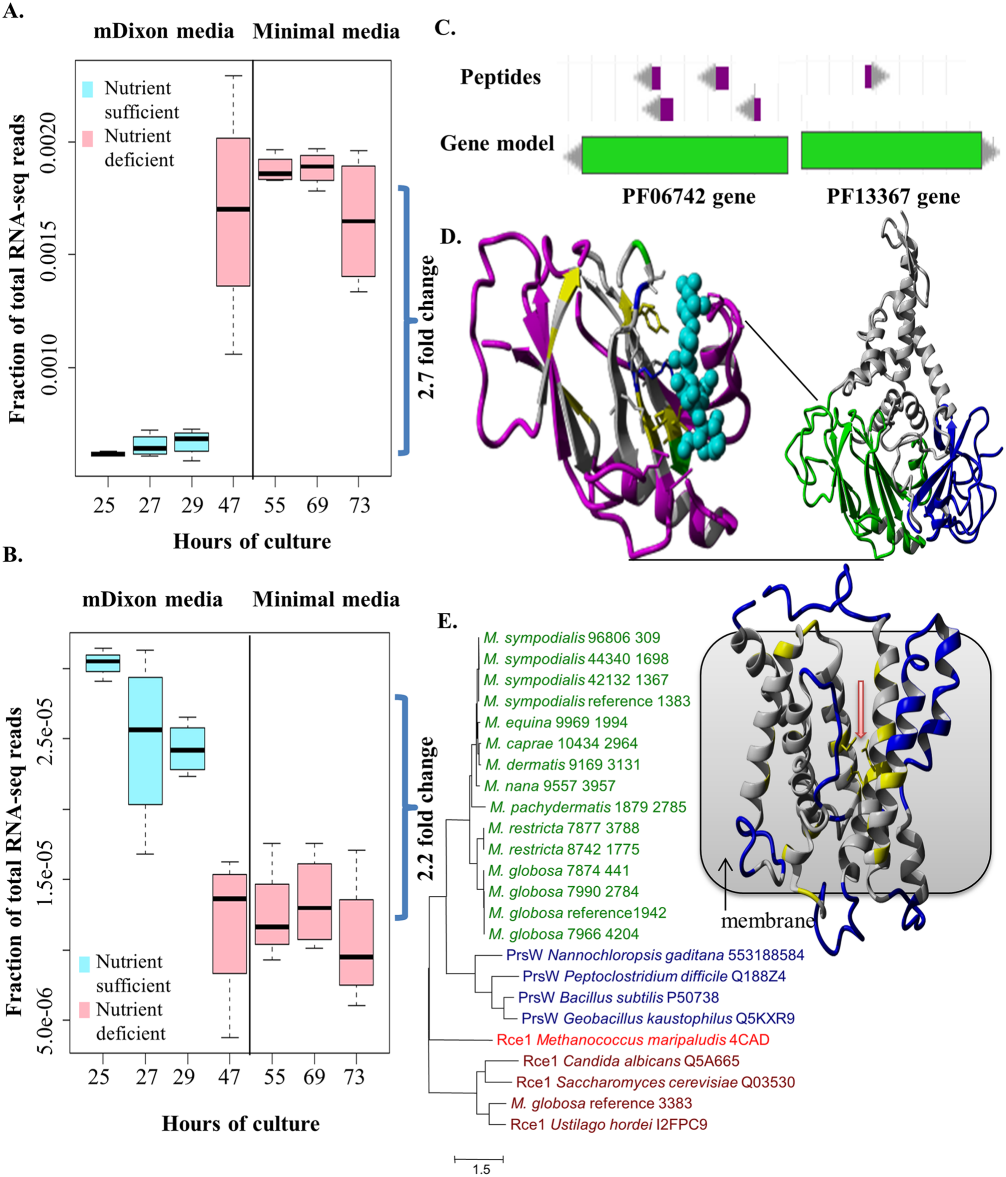
Functional characterization of novel, putative horizontally transferred genes in *Malassezia*. A) Upregulation of the gene containing the PFam domain PF06742 in nutrient deprived conditions in *M*. *globosa*. B) Downregulation of the gene containing PF13367 in nutrient deprived conditions in *M*. *globosa*. C) Peptide evidence for the genes containing PF06742 and PF13367 in *M*. *sympodialis*. D) Structural model of representative gene containing PF06742 (MGL_833 from *M*. *globosa* 7966 reference). Right side: full model with domains in different colors. Left side: zoom to Jelly Roll domain with predicted glycosyl hydrolase function (Coloring shows relative similarity to known hydrolase enzymes. Purple: structurally different; Gray: structurally same, amino acid different; Yellow: structurally same, amino acid identical, hydrophobic; Blue, Red, Green: structurally same, amino acid identical, non-hydrophobic [blue: positive charge; red: negative charge; green: polar]; Cyan: substrate sugar). E) Left side: Maximum likelihood phylogenetic tree of the *Malassezia* PrsW-like family (green) with representatives from PrsW (blue) and Rce1 (brown) families including one resolved structure (red). Gene IDs are specified behind species names and strain IDs. Right side: homology model of a PrsW-like protease (MG7966_4204 from *M*. *globosa* 7966; contains the PFam domain PF13367) with red arrow indicating conserved glutamates and histidines coming together to form the active site. Coloring: Blue: structurally different; Gray: structurally same, amino acid different; Yellow: structurally same, amino acid identical (all types).

We further investigated the role and function of three specific gene families that likely represent key gain-of-function events in *Malassezia*. The first of these is unique as it is the only one found to be conserved in all *Malassezia*. This gene family is defined by matches to the PFam domain PF06742 (a domain of unknown function) and is present in a single gene copy in all *Malassezia*, except for the *M*. *furfur* hybrids and *M*. *slooffiae* which have two gene copies. Its universal presence in all *Malassezia* and absence in all other Basidiomycetes suggests that a lateral gene transfer event in the ancestor of all *Malassezia* is the most parsimonious explanation. In addition, while the likely source of this gene could not be determined due to its ancient origin, we noted that it is seen in diverse and often pathogenic bacteria (*e*.*g*. *Mycobacterium tuberculosis*, *Listeria monocytogenes* and *Salmonella enterica*) and fungi (e.g. *Aspergillus flavus*) and is surprisingly well conserved (http://pfam.xfam.org/). Furthermore, we noted that the gene in *M*. *globosa* is significantly up-regulated in nutrient deficient conditions (Fig 4A) while its ortholog in *Chlamydomonas reinhardtii* is dramatically up-regulated under sulfur depletion conditions (http://tinyurl.com/nyjd3md), suggesting that they might serve an essential biological role. Proteomics evidence from *M*. *sympodialis* [14] indicates that this gene is likely translated (Fig 4C) and secreted (based on a signal peptide match). Homology modeling predicted its likely function to be a glycosyl hydrolase (EC 3.2.1.x, www.genome.jp/kegg/) (Fig 4D, S7 Text). The exact substrate remains to be determined but based on structural considerations there is slightly higher similarity to beta-galactosidases or mannosidases near the predicted substrate binding site (S7 Text). In addition, hydrolyzing activity on fungal cell wall glucans, which have been determined to be mainly (1->6) beta-D-glucans in *M*. *sympodialis* [36], cannot be excluded based on profile sequence searches (S7 Text). Adding to the functional context, co-expression analysis in *M*. *globosa* revealed that this putative hydrolase gene’s expression is highly correlated with that of an aspartyl protease (mgl_641, Pearson Correlation = 0.955, FDR = 2.16×10^−20^). Interestingly, in another fungus, *Candida glabrata*, an aspartyl protease is required for pH-change-induced reduction in total beta-glucan levels in the cell wall [37] which could be achieved by coordinating with a beta-glucan hydrolase. Further experimental work should help clarify this hypothesis and the gene’s impact on *Malassezia* biology.

The second gene family, with a match to the PFam domain PF00199, likely represents a case of inter-kingdom gene transfer (from bacteria) of a catalase gene whose product carries out the key function of removing the reactive oxygen species H_2_O_2_ [38]. Intriguingly our phylogenetic analysis suggests that while, in general, all *Malassezia* have one catalase that is more closely related to bacterial catalases (from *Blastomonas* and *Sphingomonas*), *M*. *slooffiae* has an additional, presumably ancestral, catalase that is more closely related to fungal catalases (S5 Fig). The acquisition of a bacterial catalase in *Malassezia* could have provided a selective advantage in adapting to life on a new host, especially considering the numerous secreted proteins (for example, GMC oxidoreductases) that could generate hydrogen peroxide [2]. Within the genus, catalase genes are missing in two species, *M*. *restricta* and *M*. *pachydermatis*, and this was confirmed by BLAST search [39] to both genomes and proteomes. For *M*. *restricta*, absence of catalase enzyme activity has been confirmed by enzyme test [40]; given the fact that *Malassezia* live in an aerobic environment on skin [1] alternative metabolic pathways might exist to detoxify oxygen in *M*. *restricta*. For *M*. *pachydermatis*, catalase activity has been observed [40] and alternative catalases might exist which are sufficiently diverged from catalases in other *Malassezia* species.

The third gene family, defined by matches to the PFam domain PF13367 (a family of putative PrsW proteases), was found to be present in all genomes of *Malassezia* cluster B (containing species commonly found on human skin) while being absent in other *Malassezia* and Basidiomycetes (S3 Table), suggesting that it may have been horizontally acquired in the lineage leading to cluster B. Genes belonging to this gene family were readily found in skin resident bacteria (e.g. *Propionibacterium*, *Streptococcus* and *Staphylococcus*) as well as a few parasitic protists (e.g. *Toxoplasma gondii*, *Neospora caninum*, *Cryptosporidium* and *Plasmodium*) (http://pfam.xfam.org/). In *Bacillus subtilis*, PrsWs sense antimicrobial peptides and then cleave the anti-Ϭ^W^ factor to activate the Ϭ^W^ factor [41]. However, in the absence of the anti-Ϭ^W^ factor or the Ϭ^W^ factor in *Malassezia*, these genes are likely to have a different role. We confirmed that this gene is expressed and translated (Fig 4B and 4C) and significantly down-regulated in nutrient deficient conditions (Fig 4B). PrsW-like proteases belong to the endopeptidase family M82 that is related to the family M79 (that includes Rce1 peptidases) (Fig 4E) [41,42] with a recently resolved crystal structure [43]. Homology modeling confirmed the known catalytically important residues [41,43] to be conserved between these two families (S6 Fig, S7 Text) and located in the center of the transmembrane bundle forming the active site (Fig 4E). The Rce1 peptidases typically cleave C-terminal tripeptides from isoprenylated proteins (e.g. fungal mating factor a) [43]. However, this is not likely the function of *Malassezia* PrsW-like family due to the presence of direct Rce1 homologs in *Malassezia* (e.g. MGL_3383, Fig 4E).

### Gene family expansion and extensive turnover underlie niche specificity in *Malassezia*

**Fig 5:**
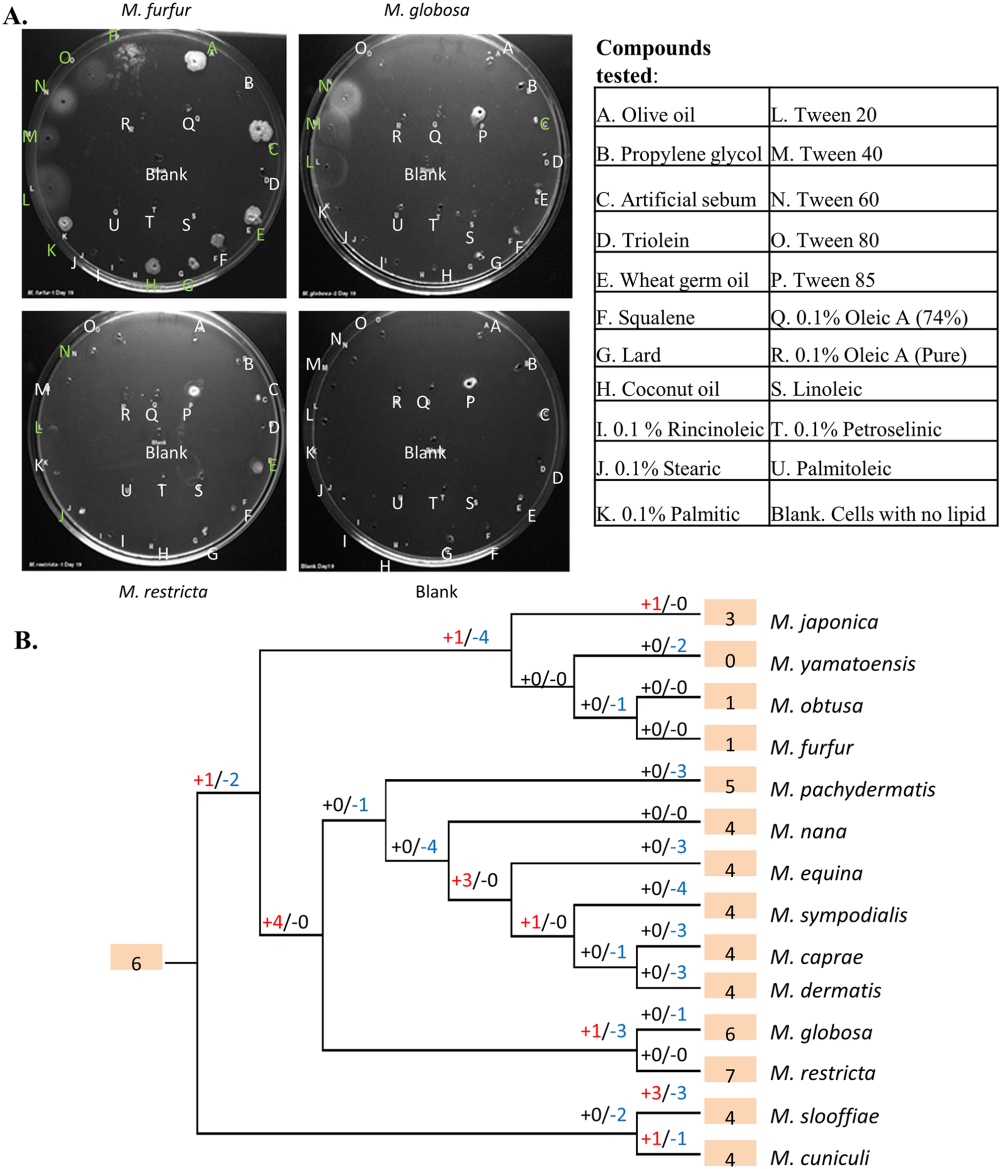
Lipid specificity and extensive turnover in the lipase gene family. A) Representative lipid assimilation assay images. Letters correspond to lipid wells (S5 Table). White letters indicate no growth, while green letters indicate growth on visual scale. B) Gene gains and losses in phosphoesterase family PF04185, where “+” indicates the number of gene gain events while “-” indicates the number of gene loss events. Shaded numbers indicate the estimated gene copy number in the most recent common ancestor and gene copy numbers in the observed species.

*Malassezia* are known to have varying host tropism and highly specific preferences for environmental niches and food sources [3,44]. For example, some highly sebaceous sites such as scalp (including occiput) and back are typically dominated by *M*. *globosa* [11]. To further understand niche-specificity in *Malassezia*, we evaluated their preference for growth in various lipid media using “Lipid Assimilation Assays” (see Methods). These experiments highlight a strong specificity in *Malassezia*’s preference for lipids (S4 Table) that is not well correlated with their phylogenetic relatedness. For example, *M*. *furfur* and *M*. *sympodialis* are functionally similar as the most robust of the lipid-dependent species in culture, sharing the broadest range of lipids that support growth (Fig 5A, S5 Table). However, they are not closely related and are placed in different sub-clusters of the *Malassezia* phylogeny (Fig 3B). Also, the closely related species *M*. *globosa* and *M*. *restricta* have different lipid assimilation profiles (Fig 5A, S5 Table). To understand the genetic basis of these phenotypes, we reexamined the list of gene family expansions and selection in *Malassezia*. Strikingly, the most expanded gene family in *Malassezia* was found to be a phospholipase family, and a secretory lipase family was also among the list of 13 families with a 2-fold increase in median copy number in *Malassezia* compared to other fungi (S3 Table). Lipase and phospholipase activities have been detected in multiple *Malassezia* species [45,46]. Their genes are highly expressed *in vivo* on human scalp [2,45,47], and are thought to play an essential role in supporting their growth. Therefore, we hypothesized that expansion of these gene families might explain *Malassezia* niche-specificity. Other than lipases, many peptidases in multiple families are found in the most expanded gene families in *Malassezia*, underlining their importance in *Malassezia* biology (S3 Table, S8 Text).

To understand the evolution of these families, we inferred parsimonious reconstructions of gain and loss events (see Methods). In particular, the most expanded gene family, a family of phospholipases (phosphoesterases, PF04185) showed a striking pattern of extensive turnover, with a dramatic expansion of the family in the ancestor of cluster B species followed by lineage specific losses (Fig 5B). Recent duplications were also observed in *M*. *japonica*, *M*. *slooffiae* and *M*. *cuniculi* while cluster A species seem to have experienced a significant contraction in this gene family that is thought to be relevant to fungal pathogenesis [48]. Extensive turnover in the lipase gene repertoire of *Malassezia* was also seen in the secretory lipases (PF03583) (S7 Fig). Species-specific duplication events were found in seven species and, in particular, there has been rapid recent expansion of the family in *M*. *slooffiae* and *M*. *pachydermatis* (S7 Fig). We observed frequent lineage-specific duplication and loss of genes in other lipase families as well (e.g. PF01764) and together these could explain the complex patterns of lipid-specificity observed in *Malassezia*. Further experiments should help establish the exact roles specific lipase genes play in the process of human colonization and pathogenesis.

## Discussion

*Malassezia*, while found on all humans and associated with many common human skin diseases, are poorly understood in large part due to a lack of genomic tools. Here, we report generation and analysis of the genomes of all 14 accepted *Malassezia* species, including multiple strains of those most commonly found on human skin (for a total of 24 strains). *Malassezia* are unique in several ways, including their adaptation to life on animal skin, their dominance as eukaryotic residents on human skin (in contrast to the diversity seen among prokaryotic commensals), and their lipid-dependent lifestyle. Even within *Malassezia*, we noted there is substantial variability in preference for food sources and thus environmental niches. As a first step, the analysis in this study serves to systematically catalog and characterize genomic features unique to *Malassezia* and its lineages, which could then be associated with the observed phenotypes. This was aided by the characterization of all known species in the genus as well as multiple strains for key species, allowing robust conclusions to be drawn despite potential analysis pitfalls. Correspondingly, several of the genes identified in this study are prime candidates for further experimental study. It is tempting to speculate, for example, that the gene containing the PFam family PF06742 serves an essential function in *Malassezia* such that loss of the gene could be lethal. As this gene was likely horizontally acquired by the ancestor of all *Malassezia*, its function could also be tied to the origin of the genus, particularly if it relates to utilizing energy sources from the host. Similarly, the role of PF13367 could be linked to the ability of cluster B *Malassezia* to thrive on human skin. In general, *Malassezia* are not facile experimental systems as they are challenging to cultivate and typically recalcitrant to genetic manipulation. In this context, recent success in performing gene deletion in *M*. *furfur* is encouraging (Giuseppe Ianiri and Alexander Idnurm, personal communication) and could enable *in vivo* functional characterization.

Among other gene families of interest, particularly due to their association with niche-specificity, are several lipase families. Interestingly, there are a total of 25 lipases found in the two major lipase families (PF03583 and PF01764) in *M*. *slooffiae*, a species found on both animals and humans [9,49] with little known about its involvement in diseases. This is the most in any haploid *Malassezia* strain (S3 Table), with the closely related *M*. *cuniculi* having only 16 lipases and *M*. *globosa* 14 (S3 Table). Many lipases in *M*. *slooffiae* are derived from unique species-specific duplication events (S7 Fig). However, it remains an open question if *M*. *slooffiae* is indeed able to leverage this large arsenal of lipases to utilize a wider range of lipids and hence live in more diverse ecosystems. Intriguingly, we observed in our skin metagenomic datasets that the only three samples with relatively high abundance of *M*. *obtusa* are in co-occurrence with *M*. *slooffiae* (Fig 2A), suggesting the possibility that *M*. *slooffiae* breaks down lipids for utilization by *M*. *obtusa*, a rare and hard-to-culture species. Further studies are needed to establish this relationship but it is clear that the availability of genomes for all *Malassezia* species will be critical to understanding their distribution and role in human diseases.

The question of whether *Malassezia* or *Malassezia-like* species are abundant in habitats other than on the skin of warm-blooded animals is an intriguing one. Our analysis of samples from varying habitats suggests that they are either not common or not similar enough to known *Malassezia* species. With the availability of a catalog of *Malassezia*-specific genes, sensitive models can be built to detect remote homologies to these sequences [29] as a way to search for distant *Malassezia-like* species in the environment. This in turn should help clarify the emergence and role of *Malassezia* as a skin-adapted fungus.

Fungal mating is hypothesized to play an important role in pathogenesis by increasing genetic diversity [32,50]. The observation that mating loci are linked in three species in cluster A and B, suggests that bipolar or pseudo-bipolar mating systems may be present in all *Malassezia*. The former is observed in most human pathogenic fungi [32] and in ascomycota, and the latter is observed in *M*. *sympodialis* [14], despite the fact that the tetrapolar mating system is more common in basidiomycota [32]. Further studies using sequencing technology which enables longer read lengths and hence analysis of larger genomic structural elements are needed to define the presence and impact of pseudo-bipolar mating systems in *Malassezia* and any role in their pathogenesis.

## Methods

### *Malassezia* culture, extraction of DNA and RNA, and library preparation

*Malassezia* was grown on mDixon media for DNA extraction, and *M*. *globosa* was grown on mDixon or minimal media for RNA extraction. Sequencing was done using Illumina HiSeq 2000. Please see S9 Text for details.

### *De novo* genome assembly

Genomes were assembled using an in-house pipeline. Specifically, we used a conservative trimming approach (removing Q2 bases and all following bases and discarding paired-end reads with one or both ends shorter than 50 bp) as recommended by Illumina. After read-trimming, *de novo* assemblies (contiging and scaffolding) were constructed using SOAPdenovo [51] (version 1.05, maximum insert length = 300bp). A range of assembly options were explored including testing various k-mers (41, 51, 61, 71, 81) and down-sampling of coverage on an exponential scale down to 50X to identify an optimal assembly in terms of contiguity statistics (N50). An example of this can be seen in S6 Table. The resulting assembly was then re-scaffolded using the program Opera version 1.4 [52] with default parameters and we attempted to close remaining gaps in the assembly *in silico* using FinIS [53]. Details of assembly statistics can be found in S7 Table.

### RNA-seq assembly for *M*. *globosa*

Illumina paired-end reads were first trimmed using fastq_quality_trimmer (-t 20 -l 10) in FASTX (http://hannonlab.cshl.edu/fastx_toolkit/). Reads that could not be paired were discarded. Then, reads were assembled into transcripts using Trinity [54] (—jaccard_clip—SS_lib_type FR). The jaccard_clip option was turned on to avoid fusion of transcripts. Assembly was done individually for each sample and all assemblies were combined to generate a complete set of 727,354 transcripts. After removing redundant transcripts that were identical to another transcript, a total of 716,864 transcripts were left. Then, transcripts that were fully contained in another transcript were also removed to generate a set of 402,757 transcripts.

### Gene annotation

The *M*. *globosa* 7966 reference genome was originally annotated by combining *in silico* prediction and a limited number of EST sequences [2]. Therefore, we re-annotated this genome to improve quality with the aid of RNA-seq datasets from two growth conditions and the application of newer *ab initio* gene prediction programs [55,56]. We used the MAKER pipeline [57] to integrate *ab initio* gene prediction (SNAP [55] and AUGUSTUS [56]), transcript evidence (from our RNA-seq dataset), and protein evidence to predict genes in an iterative manner. For protein sequences, we downloaded several fungal genomes from Genbank, including *Saccharomyces cerevisiae* [58], *Ustilago maydis* [59], *Candida albicans* [60], *M*. *globosa* [2], and *M*. *sympodialis* [14]. The protein sequences of *M*. *globosa* and *M*. *sympodialis* were not used in their own annotation process, respectively. Four sets of annotation were generated using the MAKER pipeline [57] in an iterative manner to improve *ab initio* gene prediction. First, MAKER was run with protein evidence and *ab initio* predictors trained with core eukaryotic genes predicted by CEGMA [20]. Then, MAKER was re-run twice additionally. For each time, *ab initio* predictors were retrained with gene models predicted by the previous run.

The first annotation was generated without transcripts. Compared to the reference annotation, this annotation retrieved longer genes and identified more exons and introns. The number of PFam domains identified in this annotation was slightly higher than that of the reference annotation, suggesting that it annotation captured more sequences with coding potential. The number of supported intron-exon junctions also increased, indicating that a large portion of the newly identified junctions were likely real (S2 Table, 1^st^ set). These results suggest that even without transcript evidence, our annotation captured more coding sequences and more complete genes. Upon addition of transcript evidence in the second set, the number of introns increased, while the number of genes decreased, likely due to the false merging of genes by transcripts spanning two genes (S2 Table, 2^nd^ set). Since the *M*. *globosa* genome is compact, with 4,223 genes (Table 1) in a 9.0 Mbp genome, false merging of genes during transcriptome assembly is likely. The number of PFam domains in the protein sequences also decreased. Therefore, we removed any transcript that overlapped with two or more gene models in order to generate the third annotation set (S2 Table, 3^rd^ set). The full set of transcripts was aligned to the *M*. *globosa* reference genome using BLAT with minimum identity of 99% [61] and any transcript that overlapped with more than one gene model were removed to generate the reduced set of transcripts. This reduced the transcript set from 402,757 transcripts to 322,251 transcripts. We also noted that very few genes exhibited evidence for alternatives isoforms (10 with two isoforms and one with four isoforms). Then, gene models in the second set that do not overlap with any gene in the third set were added to generate the fourth annotation set (S2 Table, 4^th^ set). Adding these transcripts led to an increased number of supported junctions, but the number of unique PFam domains and total PFam domains did not increase further (S2 Table), suggesting that *ab initio* prediction is sufficient to capture sequences with coding potential. For genomes other than *M*. *globosa* 7966, we used the same iterative approach for annotation without transcript evidence.

### Phylogenetic analysis

Species trees were constructed using the concatenated sequences of 164 core eukaryotic genes (CEGs) predicted by CEGMA [20] that are present in all *Malassezia* genomes, *U*. *maydis* genome, and the *S*. *cerevisiae* genome, and the *Malassezia* sequences are at least 90% the length of their *S*. *cerevisiae* orthologs. Sequences were aligned using MUSCLE [62] and the phylogeny was constructed using maximum likelihood (ML) approaches as implemented by RAxML [63]. RAxML was run using “–f a –m PROTGAMMAJTT” with 400 bootstraps. (Ustilaginomycotina tree was built using the same approach.) To test the robustness of our *Malassezia* phylogeny beyond bootstrap values, we applied a Bayesian approach on the same concatenated sequences using MRBAYES [64]. MRBAYES was run using “prset aamodelpr = mixed” and “mcmc nchains = 1 ngen = 300000”. We also merged individual ML gene trees for CEGs into a supertree [65]. Individual gene trees were constructed using the same approach and they were merged into a supertree using Clann [65]. Using this approach, more genes could be incorporated into the final tree, as missing a strain in a gene tree could be tolerated. Both approaches yielded the same phylogeny as the concatenated ML tree (Bayesian tree in S3A Fig). We also generated a species tree derived from the mitochondrial genomes (Jack Kennell, personal communications, S3B Fig). Despite differences observed on an intra-species level within the *M*. *furfur*, *M*. *globosa*, and *M*. *sympodialis* lineages, we found that the two trees correspond perfectly on an inter-species level.

For *M*. *furfur*-specific tree (S1 Fig), MCL clusters (described later) with two genes in each of the hybrids (*M*. *furfur* 7710, *M*. *furfur* 1878, *M*. *furfur* 4172, and *M*. *furfur* 7019) and one gene in each of the haploids (*M*. *furfur* 7982 and *M*. *furfur* JPLK23) were used. Nucleotide sequences were used instead of amino acid sequences. The genes from hybrid strains were separated into two groups based on their similarity to the genes of the two haploid strains as measured by BLAST bitscore [39]. Only clusters with less than 5% of total alignment as indels were used to minimize the effect of assembly and annotation errors. A total of 1,306 clusters were concatenated and aligned using MUSCLE [62]. To generate a maximum likelihood tree, RAxML was used with “-f a -# 400 -m GTRGAMMA” [63].

### Gene family analysis

PFam domains [29] were identified in all *Malassezia* strains and other fungal protein sequences using hmmscan (HMMER 3.1b1) with trusted cutoffs (http://hmmer.org). *Malassezia*-specific PFam domains are defined as present in at least seven *Malassezia* species and in at most two other fungi. This approach is limited to the study of protein families having at least one PFam domain. MCL clustering [66] was used to cluster *Malassezia* and other fungal genes based on their pairwise sequence similarity to construct gene families. This approach does not rely on PFam and can include all genes regardless whether they have PFam domains or not. Yet, unrelated genes with no orthology can be clustered together if they both have a good match to a third gene. BLAST [39] was used to align *Malassezia* and other fungal protein sequences in an all-against-all fashion with an e-value cutoff of 10^−5^. MCL was used to cluster protein sequences based on their *E*-values with–I 2.0. Sequences shorter than 90% of the median length in the cluster were excluded. *Malassezia*-specific clusters were defined as present in at least seven *Malassezia* species and not present in other fungi). To infer gain and loss in PFam families, PFam family phylogenies were constructed using MUSCLE and RAxML as described earlier. Then, species phylogeny and gene family phylogenies were reconciled using NOTUNG [67] to infer gain and loss events that took place along the *Malassezia* phylogeny.

For selection tests, we choose singleton PFam families in *Malassezia* species. Protein sequences were first aligned using MUSCLE (described earlier) and nucleotides were then substituted back in the sequences. PAML [68] was used for selection tests (CODEML, M7 M8 mode), and genes with bonferroni-corrected *p*-value < 0.05 were tabulated (χ^2^ test, two degrees of freedom).

### *Malassezia* and lipid dependence

For *Malassezia* growth in 2X YNB media, cultures were incubated in 2X YNB at 31°C for 24, 48, 72, and 144 hours. Four strains, *M*. *furfur* 7982, *M*. *sympodialis* 42132, *M*. *pachydermatis* 1879 and 7550 (also named by CBS, http://www.cbs.knaw.nl/), were used. For each strain, two cultures, one with and one without Tween 40 at 1%, were included in this experiment.

Two separate methods were used to extract lipids from the Bactopeptone media. Please refer to S9 Text for details.

### Lipid assimilation assays

Briefly, Sabouraud broth was melted with 3% Sea Plaque GTG (low-melt) agarose and equilibrated to 45°C. *Malassezia* cells were counted and diluted to 1x10^5^ cells/ml in Sabouraud broth with chloramphenicol, and equilibrated to 31°C. Cell suspension (30ml) and melted agar broth (30ml) were quickly mixed and poured into 150 mm dishes. Once solidified, 18 holes were made with a 2 mm punch biopsy. To each hole, 5 μl of test compound was added and the plates were incubated for 17 days at 34°C. Test compounds are listed in S5 and S6 Tables. Please refer to [69,70] for artificial sebum.

### *MAT* loci analysis

We examined the two mating type loci (*P/R* and *HD*) for all *Malassezia* species. We used the gene sequences from the reference strains for *M*. *sympodialis* and *M*. *globosa*, as queries to BLAST [39] against the genome assemblies of all *Malassezia* species to identify the locations of the *MAT* loci in these genomes. The alignments of *MAT* loci between *Malassezia* species were generated through WebACT (http://www.webact.org/WebACT/generate) using blastn with an *E*-value cutoff of 0.0001.

### Horizontal gene transfers in *Malassezia*

*M*. *sympodialis* 42132 protein sequences were compared to UniProtKB v2015_02 [71] and the NCBI Nucleotide Collection database using NCBI Blast [72]. Proteins with the most significant BLAST hit against bacteria in both databases were analyzed further. Bacterial and fungal homologs of each HGT candidate were retrieved from GenBank [73]. Multiple sequence alignments were generated using MUSCLE v3.8.31 [62]. Poorly aligned or divergent regions in the alignments were identified and excluded from further analysis using Gblocks v0.91 (with options –u = y –t = p) [74]. Phylogenetic trees and 1000 bootstraps for each alignment were generated using PhyML v20120412 (with options –m JTT –d aa) [75]. Phylogenetic trees were manually inspected and candidate horizontally transferred genes were chosen, if they were closer relatives to their bacterial orthologs than their fungal orthologs.

### *Malassezia* profiling from shotgun metagenomics datasets

We used PathoScope 2.0 [76] to estimate the abundances of the 14 *Malassezia* species in different shotgun metagenomics datasets (strain 7982 used for *M*. *furfur*, strain 7877 for *M*. *restricta*, strain 7966 for *M*. *globosa*, and strain 42132 for *M*. *sympodialis*). Before mapping to the reference genomes, the reads were filtered against a set of non-*Malassezia* fungal genomes (all fungal genomes from ftp://ftp.ncbi.nlm.nih.gov/genomes/Fungi/ plus *Ustilago maydis* [59]) using PathoScope 2.0's MAP module. To further account for potential false positives from PathoScope analysis, *Malassezia* genomes were divided into 1 kb bins and a species was considered present only if >10% of the bins were covered by at least one read. Abundances of *Malassezia* species were renormalized after removing false positives using this filter. The performance of PathoScope 2.0 was benchmarked by mapping the original reads of each *Malassezia* strain to the genomes of all *Malassezia* species using the protocol described here.

Accession numbers for the datasets studied in this paper are summarized in S9 Text.

To identify genes with variable copy number in *Malassezia*, we focused on *M*. *restricta* (using *M*. *restricta* 7877 genome), the most abundant species in skin samples [12]. We selected six samples with highest *M*. *restricta* genome coverage (MET0202, MET0207, MET0259, MET0270, MET0276, MET0278) [12]. Read counts for each gene were obtained from PathoScope [76] and normalized across all genes in each sample. Normalized read counts were used to compute the coefficient of variation (= sample standard deviation divided by sample mean). The top 10 genes with highest coefficient of variation are shown in Fig 2B and the top 15 are listed in S3 Table.

## Supporting Information

S1 Fig Phylogeny of *M*. *furfur*. MF stands for *M*. *furfur*. The upper clade includes MF7982 and the haploids within the diploid *M*. *furfur* hybrids that are more similar to MF7982. The lower clade includes MFJPLK23 and the haploids within the diploid *M*. *furfur* hybrids that are more similar to MFJPLK23.
(TIF)

S2 Fig Benchmarking metagenomic analysis pipeline. Genomic reads from each strain (x-axis) were mapped to selected genome assemblies (one genome per species, see Methods). On y-axis, percentage of reads mapped to each genome is shown in (A), indicating most genomic reads are mapped to the correct species (sensitivity); relative abundance of total mapped reads is shown in (B), indicating that after filtering with genomic bins (see Methods), our pipeline is highly specific.
(TIF)

S3 Fig *Malassezia* phylogenies generated with alternative approaches. A) Bayesian approach; B) mitochondria gene-based (only nine *Malassezia* species, branch length not to scale). Numbers indicate bootstrap values.
(TIF)

S4 Fig *MAT* loci in *Malassezia*. **A)** *MAT* loci linkage in *Malassezia*. Strains in which the linkage between the mating type *P/R* and *HD* loci has been confirmed are indicated with a “*”. The red “*”s indicate the strains in which the linkage between the *P/R* and *HD* loci are likely established through a single common event (the red star); the blue “*” indicates the linkage between *P/R* and *HD* loci in *M*. *yamatoensis*, which is likely established independently, based on the different configuration of the two *MAT* loci, as well as the enlarged, and highly diverged chromosomal region between the *P/R* and *HD* loci in this species. B) Comparison of chromosomal regions encompassing the *P/R* and *HD* loci in different *Malassezia* species. Shown here are alignments of the chromosomal regions encompassing the *P/R* and *HD* loci in the genomes of the four isolates in which the two *MAT* loci are linked. Blue lines connect homologous regions with same orientation and red lines connect homologous regions with opposite orientations. Block arrows indicate genes located within the *P/R* and *HD* (*bE* and *bW*) loci.
(TIF)

S5 Fig Phylogeny of catalase gene family (PF00199). A) phylogeny including *M*. *globosa* and *M*. *slooffiae* catalases, closely related bacterial catalases, and other fungal catalases. Red letters indicate *Malassezia* catalases; green shaded area includes three *Malassezia* catalases and their close bacterial relatives; B) phylogeny only including *Malassezia* catalases.
(TIF)

S6 Fig Multiple Sequence alignment using MAFFT E-INS-I for *Malassezia* proteins with PF13367 domains. Sequences are aligned to PrsW and Rce1 representatives (including structure entry 4cadF). Only one conserved region is shown that harbors the characteristic fully conserved “EE” and “H” motifs (pink arrows) as well as additional “E” and “H” conserved only among *Malassezia* genes with PF13367 and PrsWs (green arrows).
(TIF)

S7 Fig Gene gains and losses in lipase family PF03583. “+” indicates number of gene gain events while “-” indicates number of gene loss events. Shaded numbers indicate estimated gene number in the most recent common ancestor and gene numbers in current species.
(TIF)

S8 Fig *Malassezia* growth in 2X YNB media. Cultures were incubated in 2X YNB at 31°C for the indicated time. Tween 40 was included at 1% in cultures shown in the right half of the panel.
(TIF)

S9 Fig Summary of interkingdom gene transfer to *Malassezia*. The bottom two are identified by presence/absence of PFam domain and the rest are identified by similarity-based approaches.
(TIF)

S10 Fig Structural models and conservation of *Malassezia* proteins with PF06742 domain. A) Domain hit regions mapped to MGL_833 (from *M*. *globosa* 7966 reference) structure model with the original PF06742 domain hit colored green and the unaligned ends from the HHpred hit in magenta using YASARA. B) comparison of models from *M*. *globosa* and *M*. *sympodialis* (magenta: structurally different, likely only approximate modeling accuracy in this region; gray: structure reliable and similar; yellow: structure reliable and amino acid physical property similar). C) Conservation pattern among all *Malassezia* members of this protein family. Evolutionary conservation is calculated with RVET over a MAFFT E-INS-I alignment and shown in CONSURF-like coloring with gradient from cyan (low) to purple (high conservation).
(TIF)

S11 Fig Distribution of enzymatic functions among 12 enzymes sharing a Jelly Roll and an Immunoglobulin-like fold. Left: all 12 enzymes; right: eight hydrolases out of the 12 enzymes.
(TIF)

S12 Fig Structural superimposition of Jelly Roll domain in *M*. *globosa* PF06742 gene model with enzymes of known function. A) comparison with beta-galactosidase, PDB:1yq2. B) comparison with beta-1,4-mannanase in complex with mannohexaose, PDB:1pmh. Coloring: Purple: structure different; Gray: structure same, amino acid different; Yellow: structure same, amino acid identical hydrophobic; Blue, Red, Green: structure same, amino acid identical non-hydrophobic.
(TIF)

S13 Fig Phylogenetic tree of selected *Malassezia* PF06742 genes, their close orthologues, and glucan hydrolases with annotated EC numbers. Shaded area includes *Malassezia* PF06742 genes and their close orthologues while the rest are glucan hydrolases. The two closest EC numbered genes are marked.
(TIF)

S14 Fig Transmembrane (TM) region classification plot of MG7966_4204 (containing PF13367) from *M*. *globosa* 7966. Comparison of the predicted TM regions with known TM types. Blue: membrane anchors; red: functional TM helices; green: SCOP Alpha helices; black: predicted TMs numbered in the query protein, which mostly grouped with the functional TM helices (red).
(TIF)

S1 Text Evolution of *M*. *furfur* strains. (DOCX)

S2 Text Lipid dependence in *Malassezia*. (DOCX)

S3 Text Mating loci in *Malassezia*. (DOCX)

S4 Text Concomitant RNAi/transposon loss in *Malassezia*. (DOCX)

S5 Text Positively selected genes in *Malassezia*. (DOCX)

S6 Text Horizontal gene transfers from bacteria to *Malassezia*. (DOCX)

S7 Text Structural modeling and function prediction for *Malassezia* genes with PFam domains PF06742 or PF13367. (DOCX)

S8 Text Gene family expansions. (DOCX)

S9 Text Supplementary Methods. (DOCX)

S1 Table Evaluation of *de novo* assemblies from this study by comparison to corresponding reference genomes. (DOCX)

S2 Table Comparison of annotation quality with and without transcriptome sequencing data. Results shown are for the *M*. *globosa* 7966 reference genome. Note that the addition of transcriptomics data (in v2, v3 and v1+v3) does not seem to improve the completeness of the identified proteome (measure by the number of PFam domains identified) but slightly improves the identification of intron-exon junctions.
(DOCX)

S3 Table Gene families in *Malassezia* and other fungi. (XLSX)

S4 Table The distance between *PR* and *HD* loci in different *Malassezia* strains. (DOCX)

S5 Table General results of *Malassezia* lipid assimilation assay. Gray area denotes “Not Determined”. The symbols +, ++ and +++ denote low but detectable, moderate, and maximal growth, while—denotes no growth.
(DOCX)

S6 Table Selected lipid assimilation assay results corresponding to Fig 5A. Well/row letters correspond to lipid well in Fig 5A. All lipids delivered in either triolien or propylene glycol (-) controls. (-) equals no growth, 1–3 indicate low to maximal growth on a visual scale.
(DOCX)

S7 Table Assembly statistics for the parameters tested for isolate MR8742. The underlined assembly was found to be the optimal one (100X coverage and k-mer = 51) based on the N50 reported (the number in parentheses is the number of contigs greater than that length).
(DOCX)

S8 Table *Malassezia* genome assembly statistics. (DOCX)

S9 Table Detailed transmembrane region analysis for complexity and hydrophobicity as well as highlighting positions of likely functional relevance within the helices. (DOCX)
