# Supplementary figures and images for: Genus-Wide Comparative Genomics of Malassezia Delineates Its Phylogeny, Physiology, and Niche Adaptation on Human Skin
Source: PLoS Genet. 2015 Nov 5;11(11):e1005614. doi: 10.1371/journal.pgen.1005614 (PMC4634964; doi:10.1371/journal.pgen.1005614)

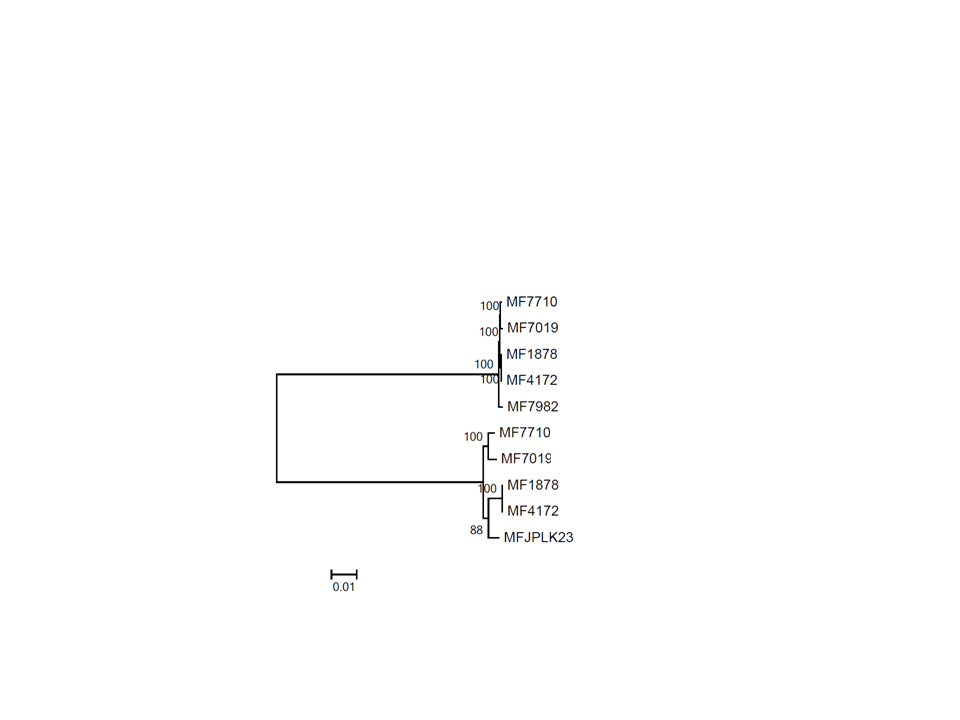

Supplement: S1 Fig — MF stands for M. furfur. The upper clade includes MF7982 and the haploids within the diploid M. furfur hybrids that are more similar to MF7982. The lower clade includes MFJPLK23 and the haploids within the diploid M. furfur hybrids that are more similar to MFJPLK23. (TIF) [file pgen.1005614.s001.tif]

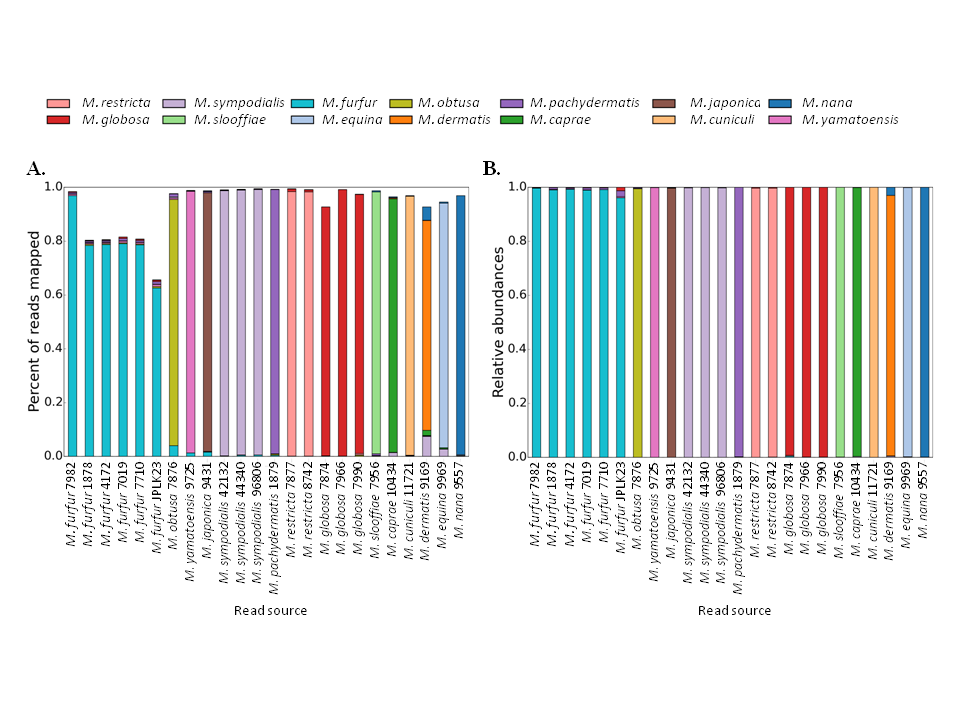

Supplement: S2 Fig — Genomic reads from each strain (x-axis) were mapped to selected genome assemblies (one genome per species, see Methods). On y-axis, percentage of reads mapped to each genome is shown in (A), indicating most genomic reads are mapped to the correct species (sensitivity); relative abundance of total mapped reads is shown in (B), indicating that after filtering with genomic bins (see Methods), our pipeline is highly specific. (TIF) [file pgen.1005614.s002.tif]

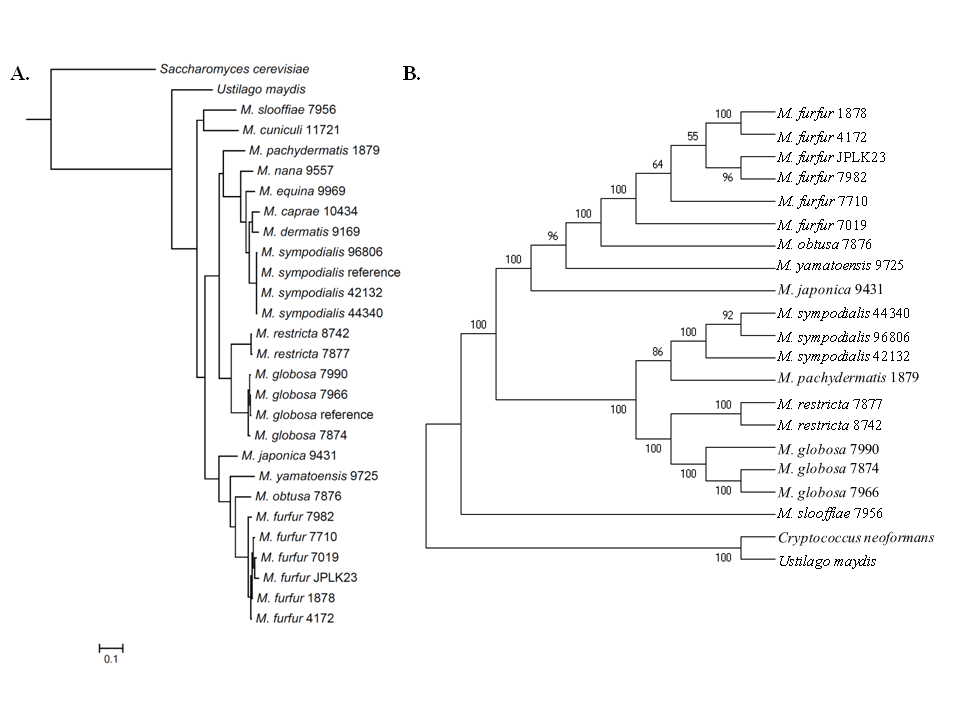

Supplement: S3 Fig — A) Bayesian approach; B) mitochondria gene-based (only nine Malassezia species, branch length not to scale). Numbers indicate bootstrap values. (TIF) [file pgen.1005614.s003.tif]

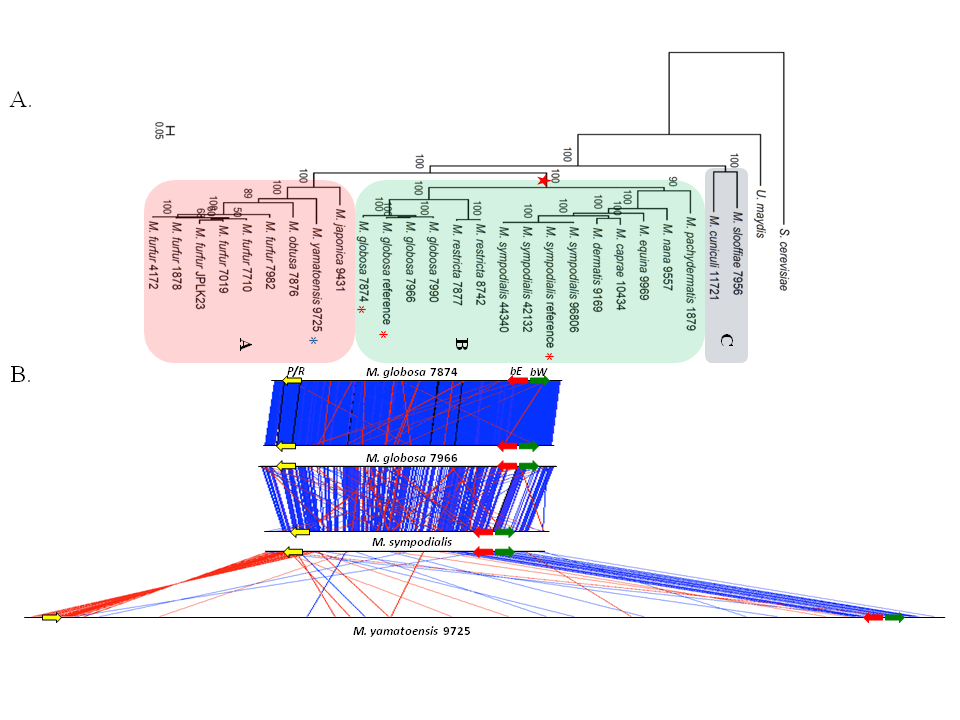

Supplement: S4 Fig — A) MAT loci linkage in Malassezia. Strains in which the linkage between the mating type P/R and HD loci has been confirmed are indicated with a “*”. The red “*”s indicate the strains in which the linkage between the P/R and HD loci are likely established through a single common event (the red star); the blue “*” indicates the linkage between P/R and HD loci in M. yamatoensis, which is likely established independently, based on the different configuration of the two MAT loci, as well as the enlarged, and highly diverged chromosomal region between the P/R and HD loci in this species. B) Comparison of chromosomal regions encompassing the P/R and HD loci in different Malassezia species. Shown here are alignments of the chromosomal regions encompassing the P/R and HD loci in the genomes of the four isolates in which the two MAT loci are linked. Blue lines connect homologous regions with same orientation and red lines connect homologous regions with opposite orientations. Block arrows indicate genes located within the P/R and HD (bE and bW) loci. (TIF) [file pgen.1005614.s004.tif]

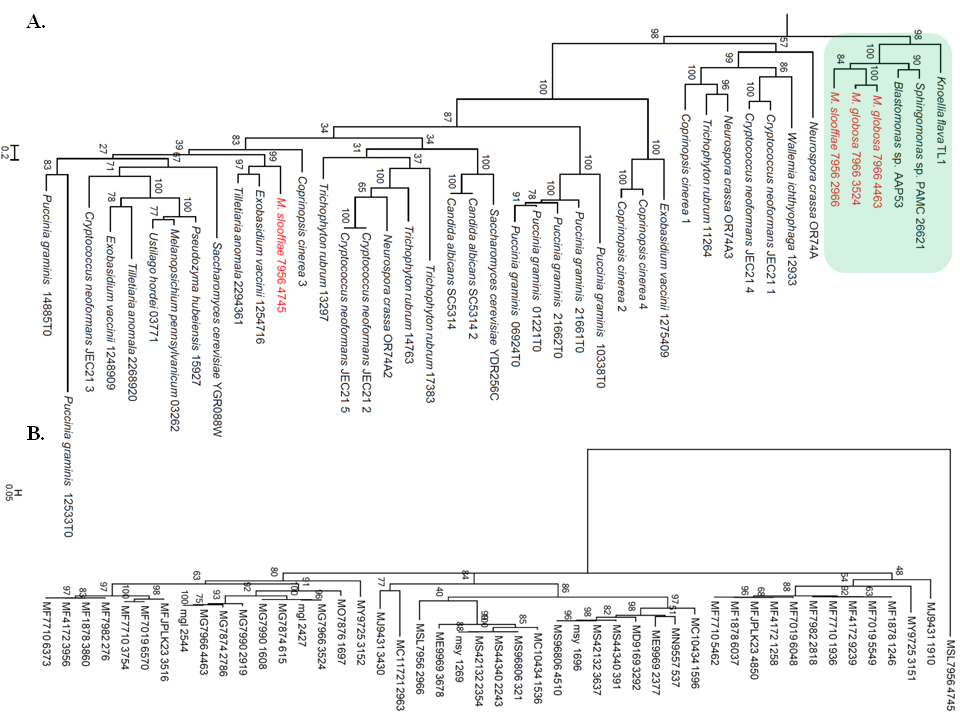

Supplement: S5 Fig — A) phylogeny including M. globosa and M. slooffiae catalases, closely related bacterial catalases, and other fungal catalases. Red letters indicate Malassezia catalases; green shaded area includes three Malassezia catalases and their close bacterial relatives; B) phylogeny only including Malassezia catalases. (TIF) [file pgen.1005614.s005.tif]

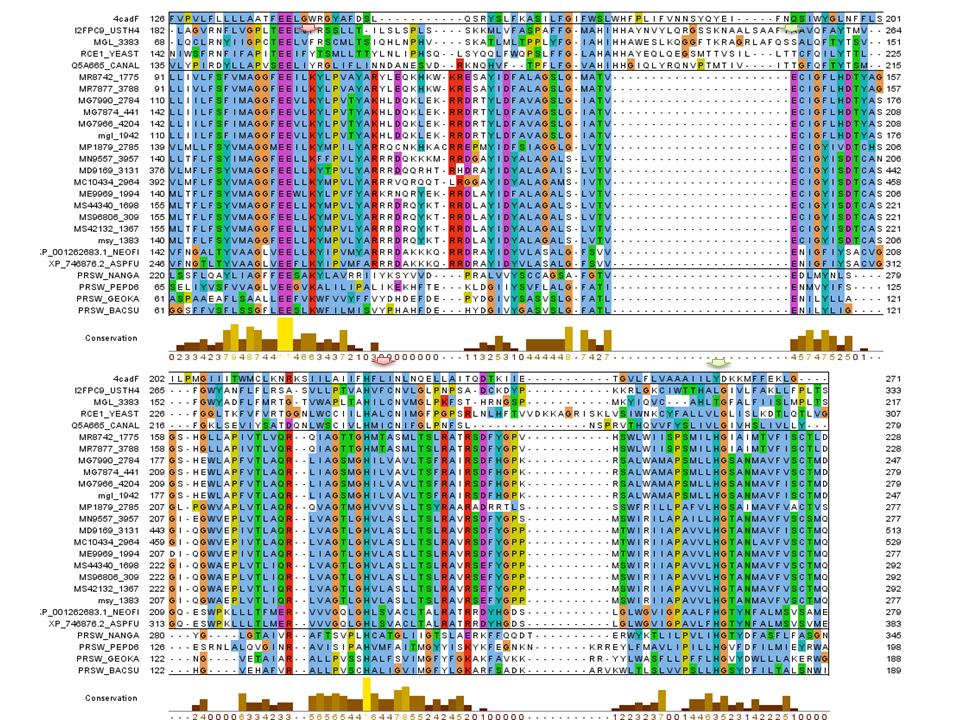

Supplement: S6 Fig — Sequences are aligned to PrsW and Rce1 representatives (including structure entry 4cadF). Only one conserved region is shown that harbors the characteristic fully conserved “EE” and “H” motifs (pink arrows) as well as additional “E” and “H” conserved only among Malassezia genes with PF13367 and PrsWs (green arrows). (TIF) [file pgen.1005614.s006.tif]

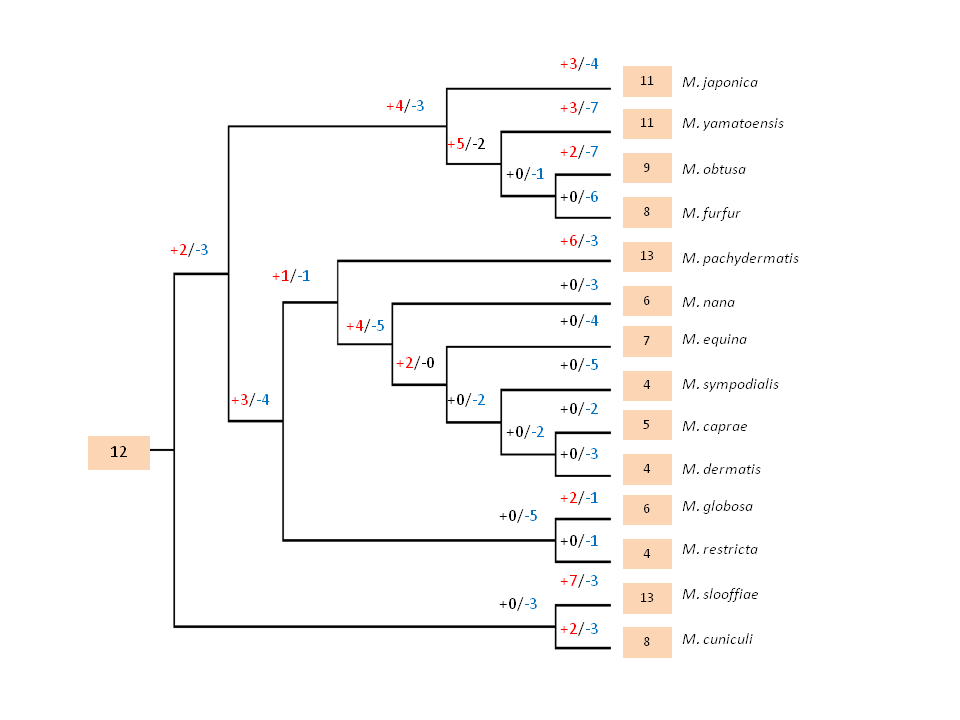

Supplement: S7 Fig — “+” indicates number of gene gain events while “-” indicates number of gene loss events. Shaded numbers indicate estimated gene number in the most recent common ancestor and gene numbers in current species. (TIF) [file pgen.1005614.s007.tif]

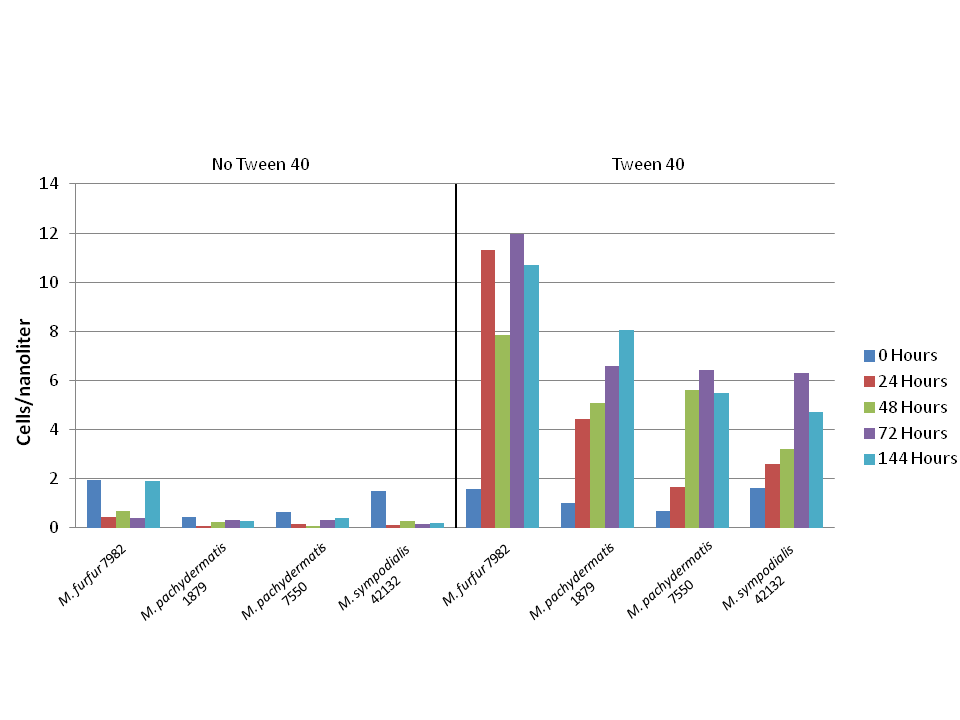

Supplement: S8 Fig — Cultures were incubated in 2X YNB at 31°C for the indicated time. Tween 40 was included at 1% in cultures shown in the right half of the panel. (TIF) [file pgen.1005614.s008.tif]

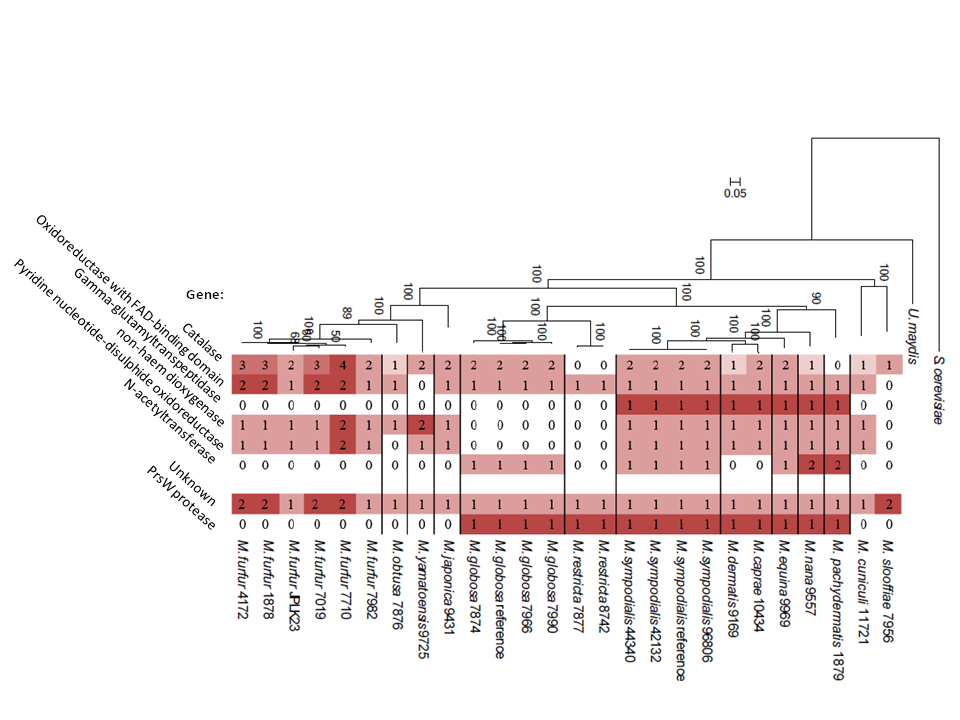

Supplement: S9 Fig — The bottom two are identified by presence/absence of PFam domain and the rest are identified by similarity-based approaches. (TIF) [file pgen.1005614.s009.tif]

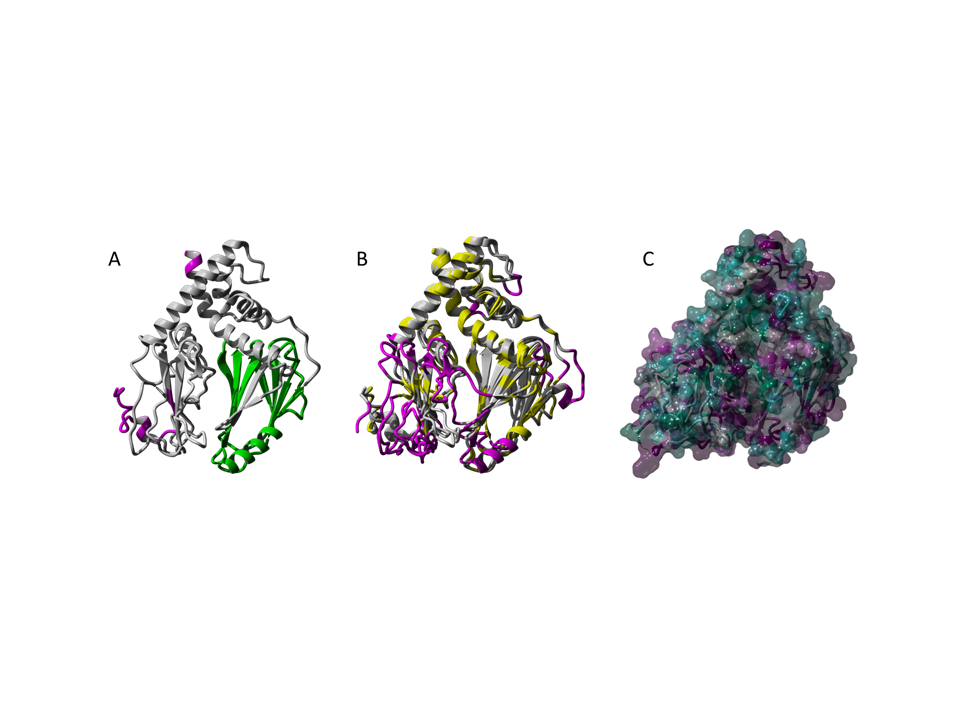

Supplement: S10 Fig — A) Domain hit regions mapped to MGL_833 (from M. globosa 7966 reference) structure model with the original PF06742 domain hit colored green and the unaligned ends from the HHpred hit in magenta using YASARA. B) comparison of models from M. globosa and M. sympodialis (magenta: structurally different, likely only approximate modeling accuracy in this region; gray: structure reliable and similar; yellow: structure reliable and amino acid physical property similar). C) Conservation pattern among all Malassezia members of this protein family. Evolutionary conservation is calculated with RVET over a MAFFT E-INS-I alignment and shown in CONSURF-like coloring with gradient from cyan (low) to purple (high conservation). (TIF) [file pgen.1005614.s010.tif]

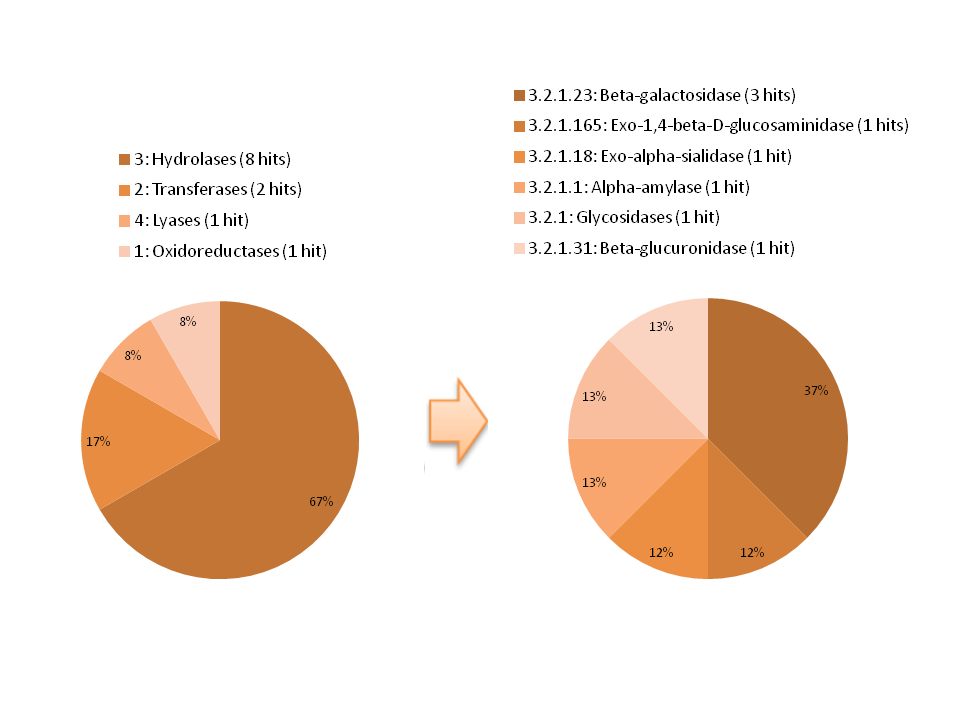

Supplement: S11 Fig — Left: all 12 enzymes; right: eight hydrolases out of the 12 enzymes. (TIF) [file pgen.1005614.s011.tif]

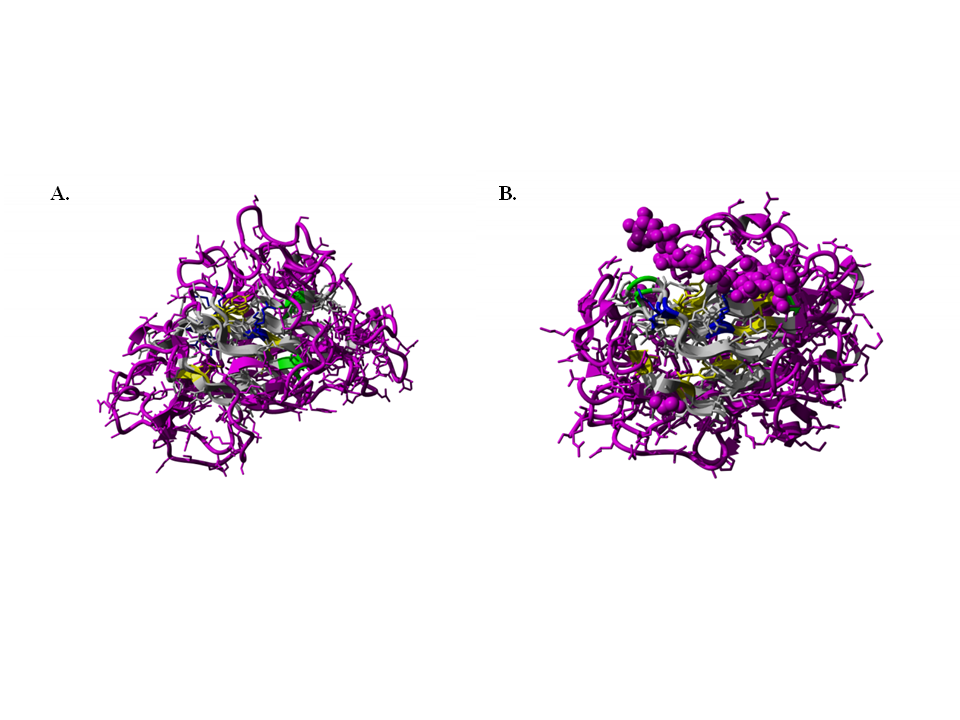

Supplement: S12 Fig — A) comparison with beta-galactosidase, PDB:1yq2. B) comparison with beta-1,4-mannanase in complex with mannohexaose, PDB:1pmh. Coloring: Purple: structure different; Gray: structure same, amino acid different; Yellow: structure same, amino acid identical hydrophobic; Blue, Red, Green: structure same, amino acid identical non-hydrophobic. (TIF) [file pgen.1005614.s012.tif]

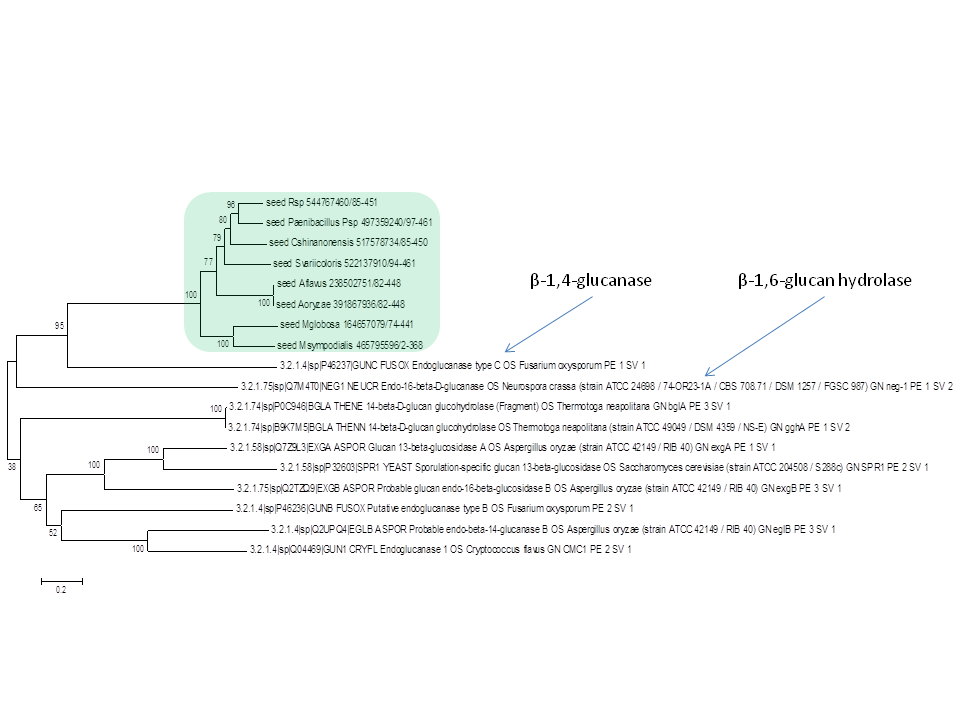

Supplement: S13 Fig — Shaded area includes Malassezia PF06742 genes and their close orthologues while the rest are glucan hydrolases. The two closest EC numbered genes are marked. (TIF) [file pgen.1005614.s013.tif]

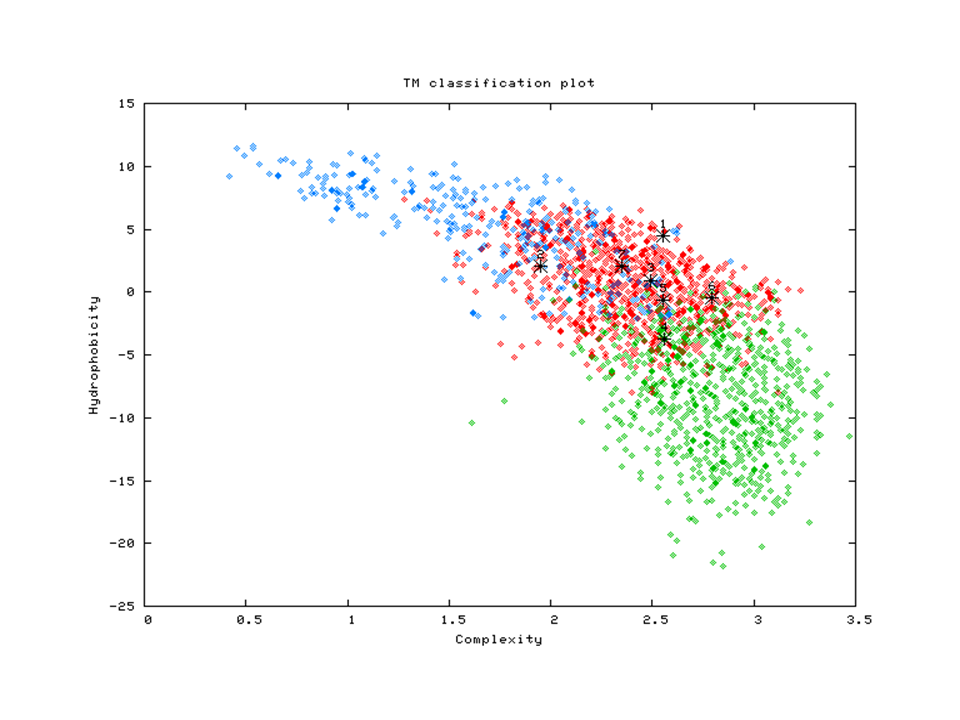

Supplement: S14 Fig — Comparison of the predicted TM regions with known TM types. Blue: membrane anchors; red: functional TM helices; green: SCOP Alpha helices; black: predicted TMs numbered in the query protein, which mostly grouped with the functional TM helices (red). (TIF) [file pgen.1005614.s014.tif]
