# Supplementary material for: Genus-Wide Comparative Genomics of Malassezia Delineates Its Phylogeny, Physiology, and Niche Adaptation on Human Skin
Source: PLoS Genet. 2015 Nov 5;11(11):e1005614. doi: 10.1371/journal.pgen.1005614 (PMC4634964; doi:10.1371/journal.pgen.1005614)
Supplement: S1 Text — (DOCX) [file pgen.1005614.s015.docx]

**S_Text 1. Evolution of *M. furfur* strains**

Some *M. furfur* strains have been found to have bigger genomes than other *M. furfur* strains using PFGE [1]. Notably, four of the strains in our study appear to have genomes (and corresponding number of genes) that are twice the average size (**Table 1**). To understand the emergence and evolutionary relationship of the *M. furfur* strains in this study, we sought to build phylogenetic trees for all strains. We based our phylogenetic analysis on nucleotide sequence comparisons as many protein sequences are identical among the strains. For each of the four unusual *M. furfur* strains, we noted that genes that were typically in single copy in other *Malassezia* were in two copies in these strains such that one copy was highly similar to the singly copy of the gene in *M. furfur* 7982 while the other copy was more similar to *M. furfur* JPLK23. Exploiting this pattern, we split the set of two copy genes into two single copy sets, essentially reconstructing haploid genomes from the potentially hybrid strains, and used these to reconstruct a multi-gene phylogeny (see **Methods**). As expected, this phylogeny further emphasizes the fact that *M. furfur* 7982 and *M. furfur* JPLK23 are distantly related and that the four unusual strains likely arose from hybridization events between these two lineages of *M. furfur* (**S_Fig 1**). These four hybrids may be intra-species hybrids or possibly inter-species hybrids if *M. furfur* is a species complex.

**References**

1. Boekhout T, Kamp M, Guého E. Molecular typing of Malassezia species with PFGE and RAPD. Med Mycol. 1998;36: 365–372.
