# Supplementary material for: Genus-Wide Comparative Genomics of Malassezia Delineates Its Phylogeny, Physiology, and Niche Adaptation on Human Skin
Source: PLoS Genet. 2015 Nov 5;11(11):e1005614. doi: 10.1371/journal.pgen.1005614 (PMC4634964; doi:10.1371/journal.pgen.1005614)
Supplement: S2 Text — (DOCX) [file pgen.1005614.s016.docx]

**S_Text 2. Lipid dependence in *Malassezia***

Previously, it was thought that *M. pachydermatis* is the exceptional *Malassezia* because it does not require added lipid for growth, though it does show enhanced growth on lipids (described as lipophilic) [1]. *M. globosa* and *M. sympodialis* were shown to lack a fatty acid synthase (FAS) gene [2,3]. Using homology analysis as described in [2], we were also unable to detect a FAS gene in the *M. pachydermatis* genome and in any other *Malassezia* genomes. The structurally similar polyketide synthases were readily found in *Malassezia* genomes (**S_Table 3**), as identified with the polyketide synthase dehydratase domain (PF14765). The *M. pachydermatis* genome harbors two copies of the polyketide synthase gene, resulting from a species-specific duplication.

We re-examined the lipid dependence of *M. pachydermatis* strains 1879 and 7550 and found that both were unable to grow in 2 X YNB defined medium in the absence of added lipid (**S_Fig 8**). As this medium supports some growth in the presence of added lipid (Tween 40), these results are consistent with the absence of a fatty acid synthase gene. It has been reported [1] that *M. pachydermatis* was able to grow on Sabouraud-dextrose agar medium, while the other species were not. We also observed growth of *M. pachydermatis* 7550 and 1879 on Sabouraud-Dextrose agar plates, and we passed these cultures on Sabouraud-dextrose agar for four additional times. The peptone in Sabouraud-dextrose medium contains 0.6% lipid [4], raising the possibility that peptone contains sufficient lipid to support growth of *M. pachydermatis*. We characterized the fatty acid content of peptone and found 6 µg of palmitic acid per gram of peptone, with lesser amounts of other fatty acids.

In summary, for the two *M. pachydermatis* strains: 1) there is no fatty acid synthase gene in strain 1879, 2) neither strain is able to grow in the defined medium 2X YNB without added lipid, and 3) both strains continue to grow upon multiple passes on Sabouraud-Dextrose agar in the absence of added lipid, presumably by using the lipid from the peptone. This supports the proposition that *M. pachydermatis* is actually lipid-dependent. These results indicate an increased efficiency in lipid uptake or metabolism, the details of which remain to be elucidated.

**References**

1. Guého-Kellermann E, Boekhout T, Begerow D. Biodiversity, Phylogeny and Ultrastructure. In: Boekhout T, MD PM, Guého-Kellermann E, Velegraki A, editors. Malassezia and the Skin. Springer Berlin Heidelberg; 2010. pp. 17–63.

2. Xu J, Saunders CW, Hu P, Grant RA, Boekhout T, Kuramae EE, et al. Dandruff-associated Malassezia genomes reveal convergent and divergent virulence traits shared with plant and human fungal pathogens. Proc Natl Acad Sci. 2007;104: 18730–18735.

3. Gioti A, Nystedt B, Li W, Xu J, Andersson A, Averette AF, et al. Genomic Insights into the Atopic Eczema-Associated Skin Commensal Yeast Malassezia sympodialis. mBio. 2013;4: e00572–12.

4. Klompong V, Benjakul S, Kantachote D, Shahidi F. Characteristics and Use of Yellow Stripe Trevally Hydrolysate as Culture Media. J Food Sci. 2009;74: S219–S225.
