# Supplementary material for: Genus-Wide Comparative Genomics of Malassezia Delineates Its Phylogeny, Physiology, and Niche Adaptation on Human Skin
Source: PLoS Genet. 2015 Nov 5;11(11):e1005614. doi: 10.1371/journal.pgen.1005614 (PMC4634964; doi:10.1371/journal.pgen.1005614)
Supplement: S3 Text — (DOCX) [file pgen.1005614.s017.docx]

**S_Text 3. Mating loci in *Malassezia***

Other than the gene repertoire, we examined the linkage in mating loci (*MAT*), which might play a role in fungal pathogenesis [1]. We found that the *bW* and *bE* genes from the *HD* locus are adjacent to each other and have divergent orientations in all *Malassezia* species. Additionally, we found evidence supporting the physical linkage between the two *MAT* loci (i.e. co-location on the same chromosome or contig) in four genomes: *M. sympodialis* reference strain (42132), *M. globosa* reference strain (7966), *M. globosa* 7874, and *M. yamatoensis* 9725 (**S_Table** **8**). It should be noted that because most of the genomes are not as yet assembled as complete chromosomes, linkage between the *P/R* and *HD* loci might be undetectable because they are still on separate contigs. The four strains belonged to two clusters (A and B, **S_Fig** **4A**). A comparison of their chromosomal regions encompassing the *P/R* and *HD* loci suggests two independent events that led to the linkage between the two *MAT* loci (**S_Fig** **4**). Specifically, the two *MAT* loci, as well as the chromosomal region in between the two *MAT* loci, are in overall synteny between *M. globosa* and *M. sympodialis* isolates, suggesting a single event gave rise to the linkage in these two species; however, compared to *M. globosa* and *M. sympodialis*, the chromosomal region encompassing the two *MAT* loci in *M. yamatoensis* 9725 is considerably larger, with no homology observed in the region linking the two *MAT* loci, and the orientation of the *P/R* locus relative to the *HD* locus is inverted in *M. yamatoensis* 9725 compared to the *M. globosa* and *M. sympodialis* (**S_Fig** **4B**), suggesting an independent event gave rise to the linkage between the two *MAT* loci in *M. yamatoensis* 9725 (**S_Fig** **4A**). Overall, we confirm linkage between the two *MAT* loci in three *Malassezia* species, which is uncommon among basidiomycetes but common among human-pathogenic fungi [1].

**References**

1. Nielsen K, Heitman J. Sex and Virulence of Human Pathogenic Fungi. In: Dunlap JC, editor. Advances in Genetics. Academic Press; 2007. pp. 143–173. Available: http://www.sciencedirect.com/science/article/pii/S006526600657004X
