# Supplementary material for: Genus-Wide Comparative Genomics of Malassezia Delineates Its Phylogeny, Physiology, and Niche Adaptation on Human Skin
Source: PLoS Genet. 2015 Nov 5;11(11):e1005614. doi: 10.1371/journal.pgen.1005614 (PMC4634964; doi:10.1371/journal.pgen.1005614)
Supplement: S4 Text — (DOCX) [file pgen.1005614.s018.docx]

**S_Text 4. Concomitant RNAi/transposon loss in *Malassezia***

BLAST databases were constructed for the 24 genomes produced in the study, and a tBLASTn search was performed using the amino acid sequence of the argonaute, RdRp, and two dicer proteins from H99 C*ryptococcus neoformans*. None of the four proteins searched produced homology with an E-value below 0.1 against any of the 24 genomes, even without adjustment for performance of multiple tests, while most hits had E-values substantially above 1. This suggests that the RNAi pathway was not present in the last common ancestor of the *Malassezia* lineage. Numerous losses of RNAi have occurred throughout the fungal and eukaryotic kingdoms, despite its role as a conserved pathway present in the putative last common ancestor of all eukaryotes [1,2]. Loss of RNAi in *Malassezia* could be the result of a shift into close association with the skin of animal hosts and a resultant change in lifestyle. Studies with additional outgroups may be necessary to determine when the actual loss occurred.

A *de novo* prediction of transposons was performed using PILER [3] on all 24 *Malassezia* genomes, as well as the two reference genomes. Both the distributed family and terminal repeat family modules were employed, but only a single family containing three members was identified from the *Malassezia pachydermatis* genome. This family showed no enrichment in the other lineages, suggesting it may be a false positive, such as a paralogous family of non-mobile elements. RepeatMasker ([www.repeatmasker.org](http://www.repeatmasker.org)) was also used to search both reference genomes for both a repeat library identified in the basidiomycete *Cryptococcus neoformans* and the default human genome mobile element library. Surprisingly, only one family of *Cryptococcus* elements matched the *Malassezia* genomes, and only at very low copy number (<5) in one location, while the human library provided more positive hits, but with no more than two elements in a 10 kb window. In some other basidiomycetes like *Cryptococcus*, the centromeres can be defined based on high density of transposable elements, but in lineages that have lost RNAi, the number of transposable elements is also typically reduced. The *Malassezia* species appear to be another example of this concomitant RNAi/transposon loss.

**References**

1. Drinnenberg IA, Weinberg DE, Xie KT, Mower JP, Wolfe KH, Fink GR, et al. RNAi in Budding Yeast. Science. 2009;326: 544–550.

2. Billmyre RB, Calo S, Feretzaki M, Wang X, Heitman J. RNAi function, diversity, and loss in the fungal kingdom. Chromosome Res Int J Mol Supramol Evol Asp Chromosome Biol. 2013;21: 561–572.

3. Edgar RC, Myers EW. PILER: identification and classification of genomic repeats. Bioinforma Oxf Engl. 2005;21 Suppl 1: i152–158.
