# Supplementary material for: Genus-Wide Comparative Genomics of Malassezia Delineates Its Phylogeny, Physiology, and Niche Adaptation on Human Skin
Source: PLoS Genet. 2015 Nov 5;11(11):e1005614. doi: 10.1371/journal.pgen.1005614 (PMC4634964; doi:10.1371/journal.pgen.1005614)
Supplement: S5 Text — (DOCX) [file pgen.1005614.s019.docx]

**S_Text 5. Positively selected genes in *Malassezia***

We identified 100 domain families under positive selection out of a total of 1,529 singleton *Malassezia* PFam families. An enrichment test revealed no significantly enriched functional categories. Therefore, we examine functions on individual domain family basis and showcase the following examples. 1) We found the domain family under the most positive selection pressure to be PF12481, an aluminium induced protein (**S_Table 3**), as is described in **Results.** 2) The domain family PF13844, a glycosyl transferase family (**S_Table 3**), is under positive selection while in general, genes involved in carbohydrate metabolism tend to be lost (**Results**), suggesting that it might play an essential role in *Malassezia* biology. 3) Calpain family cysteine proteases (PF00648), ubiquitously found in mammals and other organisms (<http://pfam.xfam.org/>), are also under positive selection (**S_Table 3**). This might suggest that these proteases are evolving fast in *Malassezia* to accommodate the need to digest various host proteins. 4) PF06333, mediator complex subunit 13 (<http://pfam.xfam.org/>), is found to be under positive selection. Interestingly, a cluster of 22 *Malassezia* genes, 12 of which contain PF06333 (**S_Table 3**), is found to be *Malassezia*-specific. Further analysis is needed for functional inference.
