# Supplementary material for: Genus-Wide Comparative Genomics of Malassezia Delineates Its Phylogeny, Physiology, and Niche Adaptation on Human Skin
Source: PLoS Genet. 2015 Nov 5;11(11):e1005614. doi: 10.1371/journal.pgen.1005614 (PMC4634964; doi:10.1371/journal.pgen.1005614)
Supplement: S6 Text — (DOCX) [file pgen.1005614.s020.docx]

**S_Text 6. Horizontal gene transfers from bacteria to *Malassezia***

It is generally accepted that horizontally transferred genes (HTGs) have had a dramatic effect on the genomes of bacteria and archaea [1]. Although the frequency is much lower in eukaryotes, several hundred transfers into fungal genomes have been identified [2,3]. The majority of identified donors are of bacterial origin [2,3]. HTGs have had a major influence on fungal metabolism, including the ability of fungi to utilize complex polymers as food sources [4], to invade plant hosts [5], to detoxify heavy metals [3] and to produce secondary metabolites [6,7]. Incidences of horizontal transfer from bacteria into *Malassezia* species were determined by identifying proteins from *M. sympodialis* 42132 that are more similar to bacterial proteins than to other fungal proteins, and then inferring their phylogenetic relationships (**S_Table 3** and **S_Fig 9**). We identified six robust gene transfers, including one first described in the *M. globosa* genome (N-acetyltransferase, NAT (MS42132_365)) [8]. The bacterial NAT gene appears to have been acquired in the ancestor of cluster B, and was subsequently lost in *M. restricta*, *M. caprae*, and *M. dermatis* (**S_Fig 9**). NAT genes have a very patchy distribution in the fungi; they are missing from Saccharomycotina species, and present in many Pezizomycotina and some Basidiomycota (predominantly the Ustilaginomycotina and the Puccinomycotina). Their biological role is not clear, though there is some suggestion that they may be involved in production of fungal secondary metabolites [8].

The five novel horizontally transferred genes include a catalase (MS42132_2354), an oxidoreductase with a FAD-binding domain (MS42132_735), a putative pyridine nucleotide-disulphide oxidoreductase (PNDR, MS42132_3641), a gamma-glutamyltranspeptidase, (MS42132_283) and a non-haem dioxygenase (MS42132_1472) (**S_Table 3)**. The bacterial origin of HTGs was identified at the taxonomic level for the FAD-binding oxidoreductase (Actinomycetales), PNDR (Dietzia) and the dioxygenase (Xanthomonadaceae).

We further investigated the distribution of the horizontal transfer events across all sequenced *Malassezia* genomes (**S_Fig 9**)**.** Some transfers are ancient - for example, the oxidoreductase MS42132_735 is significantly different to other fungal oxidoreductases, and is present in all analyzed *Malassezia* species except *M. yamatoensis* and *M. slooffiae*. The horizontal transfer event most likely occurred in the ancestor of all *Malassezia*, and was independently lost in the latter two. The acquisition of the catalase (MS42132_2354) is also likely an ancient event, because it is found in most *Malassezia* species. The catalases in *Malassezia* cluster together (except for one from *M. slooffiae*), and are more closely related to bacterial catalases than to other fungal catalases (**S_Fig 5**). One *M. slooffiae* catalase is related to fungal catalases, and probably represents the ancestral enzyme (**S_Fig 5**). Catalases are missing in two species, *M. restricta* and *M. pachydermatis* (**S_Fig 9**), which likely represent two independent gene loss events. Acquisition of catalase genes as HTGs has been described previously in three fungal pathogens of plants [3], and in the microsporidian *Nosema locustae* [9].

Interestingly, it is likely that there have been several independent losses of HTGs in *M. restricta* and *M. globosa*. Both the dioxygenase MS42132_1472 and the PNDR MS42132_3641 were probably acquired in the ancestor of all *Malassezia*, and then lost in the lineage leading to *M. globosa* and *M. restricta*, and independently lost in *M. slooffiae* (**S_Fig 9**)**.** In contrast, acquisition of MS42132_283 is likely to be a more recent event, as it is found only in *M. sympodialis* and its closest relatives, *M. dermatis*, *M. caprae*, *M. equine*, *M. nana* and *M. pachydermatis* (**S_Fig 9**). The acquisition of this transpeptidase may enable detoxification of xenobiotics.

We also identified HTGs based on PFam domain presence in *Malassezia* and absence in other fungi (see **Results**). Two examples of such genes are described in greater detail in the **Results** section, one of which was found to be present in the entire *Malassezia* genus and the other was found in all species in cluster B (a large cluster with eight species) (**S_Table 3**). In addition, interesting examples of less wide-spread lineage-specific gene gains (likely HTGs) include rubrerythrin, a gene involved in oxidative stress protection [10], that is only found in cluster C (two species) and peptidase family M23 that is only found in *M. furfur* in cluster A (**S_Table 3**).

**References**

1. Koonin EV, Wolf YI. Evolution of microbes and viruses: a paradigm shift in evolutionary biology? Front Cell Infect Microbiol. 2012;2: 119.

2. Richards TA, Leonard G, Soanes DM, Talbot NJ. Gene transfer into the fungi. Fungal Biol Rev. 2011;25: 98–110.

3. Marcet-Houben M, Gabaldón T. Acquisition of prokaryotic genes by fungal genomes. Trends Genet TIG. 2010;26: 5–8.

4. Richards TA, Talbot NJ. Horizontal gene transfer in osmotrophs: playing with public goods. Nat Rev Microbiol. 2013;11: 720–727.

5. Soanes D, Richards TA. Horizontal gene transfer in eukaryotic plant pathogens. Annu Rev Phytopathol. 2014;52: 583–614.

6. Khaldi N, Collemare J, Lebrun M-H, Wolfe KH. Evidence for horizontal transfer of a secondary metabolite gene cluster between fungi. Genome Biol. 2008;9: R18.

7. Slot JC, Rokas A. Horizontal transfer of a large and highly toxic secondary metabolic gene cluster between fungi. Curr Biol CB. 2011;21: 134–139.

8. Martins M, Dairou J, Rodrigues-Lima F, Dupret J-M, Silar P. Insights into the phylogeny or arylamine N-acetyltransferases in fungi. J Mol Evol. 2010;71: 141–152.

9. Fast NM, Law JS, Williams BAP, Keeling PJ. Bacterial catalase in the microsporidian Nosema locustae: implications for microsporidian metabolism and genome evolution. Eukaryot Cell. 2003;2: 1069–1075.

10. Sztukowska M, Bugno M, Potempa J, Travis J, Kurtz DM. Role of rubrerythrin in the oxidative stress response of Porphyromonas gingivalis. Mol Microbiol. 2002;44: 479–488.
