# Supplementary material for: Genus-Wide Comparative Genomics of Malassezia Delineates Its Phylogeny, Physiology, and Niche Adaptation on Human Skin
Source: PLoS Genet. 2015 Nov 5;11(11):e1005614. doi: 10.1371/journal.pgen.1005614 (PMC4634964; doi:10.1371/journal.pgen.1005614)
Supplement: S7 Text — (DOCX) [file pgen.1005614.s021.docx]

**S_Text 7. Structural modeling and function prediction for *Malassezia* genes with PFam domains PF06742 or PF13367**

*PF06742*: There are at least two lines of evidence leading to the same structure prediction for *Malassezia* genes with PF06742 domains such as MGL_833 (in *M. globosa* reference genome), which are found in all *Malassezia* species. First, a significant HMMER [1] hit to PFam [2] family PF06742 (from position 123 to 221 with E-Value of 0.0054) which has members with known structure but unknown function (3 PDB structures of 2 proteins from *Vibrio parahaemolyticus*). Second, direct and longer significant hits to the above mentioned structures with HHpred [3] (from position 56 to 395 with *E*-value of 4.7e-26 for PDB:3u07|A and from position 25 to 395 with *E*-value of 1.3E-24 for PDB:2p3y|A). Homology models (**S_Fig 10A** and **B**) were created using MODELLER [4] based on the above identified two significant templates for the PF06742 domain family representatives from *M. globosa* and *M. sympodialis*, respectively (5 repeats each and best model determined by DOPE score quality check).

The domain hit of PF06742 in MGL_833 (green in **S_Fig 10A**) is in a region defined by a prominent beta sandwich substructure called Jelly Roll as seen in all PF06742 members with known structure (e.g. PDB:2p3y). The Jelly Roll structural family (CATH 2.60.120) is quite common (>800 members) in the CATH structure classification [5] and its members have many different functions. Among those that are enzymes the most common role is that of hydrolases (>60%). Among the hydrolases with Jelly Roll structure, the most common functional representatives are glycosylases (>70%, 369 hits). Binding and acting on sugars would be in agreement with extracellular localization and fungus host interaction scenarios. Alternatively, hydrolases acting on lipids would also be possible in this context.

We also analyzed evolutionary conservation of residues in this domain family across the *Malassezia* genus using MAFFT [6] and RVET [7] and mapped the conservation to the structural model visualized in YASARA [8]. We see that only a few patches on the surface are conserved with some prominent conservation in the top region of the modeled helical linker domain as well as in the interface between the Jelly Roll and Sandwich domain which could hint to being an entry to the predicted sugar substrate binding pocket (**S_Fig 10C**).

Since there are still too many different functions possible (there are many proteins with Jelly Roll folds), we next tried to look only at known functions of proteins that share further domains with the *Malassezia* MGL_833 protein family. In particular, the uncharacterized proteins from *Vibrio parahaemolyticus* with PF06742 domain crystallized by a structural genomics consortium have an additional PF06863 domain (also of unknown function) at the C-terminus which also is predicted to be present in the *Malassezia* homologues based on the HHpred structure prediction. This second domain has an immunoglobulin-like beta sandwich fold (CATH 2.60.40) and is similar to the Jelly Roll but smaller. A CathTree search for proteins with both Jelly Rolls and immunoglobulin-like folds limited to representative structures at 40% sequence identity finds 22 hits of which 12 have annotated enzymatic functions. Eight of these 12 are hydrolases acting on carbohydrates (**S_Fig 11**) with the shared enzyme code of 3.2.1.x which would hence represent our function prediction for this domain.

Although inferring further details in the function prediction is difficult, we evaluated the structures of the above mentioned eight hydrolases by pairwise superimpositions with each other. Our models consider that identical or similar hydrophobic residues in structural superimposition would be important for the fold rather than function, while given the same fold, the same function could be related to the number of identical non-hydrophobic residues, especially when close to ligands. Based on these structural considerations, we shortlist two more detailed candidate function predictions, beta-galactosidase (EC 3.2.1.23) and beta-1,4-mannanase (EC 3.2.1.78). Their known structures showed slightly higher similarity to our models in the predicted binding pocket residues, especially including a prominent positive charge that is important for interaction with the carbohydrate ligand (**S_Fig 12**). While this critical arginine residue is not generally conserved in bacterial members of the PF06742 domain family, it is invariant within the *Malassezia* proteins we identified here.

However, the above workflow of narrowing down to possible functions is strongly limited by the number of available structures for the respective enzymatic reactions. At the same time, direct searches in linear sequence space (e.g. BLAST) or known domains (e.g. PFam) do not provide any useful hits. Therefore, we tried more powerful customized sequence profile searches and generated a non-redundant profile alignment of the region corresponding to the above discussed Jelly Roll domain from close hits of our *Malassezia* query protein and searched it with HMMER3 [1] (hmmscan) against the UniProtKB/SwissProt database as source for more functionally annotated protein sequences. Although there was no immediate significant hit (E<0.1), the best hit against a fungal sequence (E=2.2) corresponded to a (1->4)-beta-D-glucanase from *Fusarium oxysporum* (EC 3.2.1.4). Therefore, glucan hydrolyzing activity could be another possible function for this domain. To further evaluate this hit and related glucan hydrolases, we collected respective annotated sequences from UniProtKB/SwissProt and recreated the profile to sequence alignment from the previous search using the MAFFT [6] seed option followed by a neighbor joining phylogenetic tree with 500 bootstrap steps using MEGA [9] (**S_Fig 13**). This confirms the closest hit among these enzymes to be the (1->4)-beta-D-glucanase hit from the previous search but also highlights a second nearest candidate, a (1->6)-beta-D-glucanase from *Neurospora crassa* (EC 3.2.1.75). This is of interest because the *M. sympodialis* cell wall has been determined to include mainly (1->6) beta-D-glucans [10].

To consider all available additional data to determine the functional context of this new gene family, we performed co-expression analysis in *M. globosa* and revealed that the putative hydrolase gene’s expression is highly correlated with that of an aspartyl protease (MGL_641, Pearson Correlation = 0.955, FDR = 2.16×10^-20^). Interestingly, in another fungal system, *Candida glabrata*, an aspartyl protease is required for pH-change-induced reduction in total beta-glucan levels in the cell wall [11] which could be achieved by coordinated action with a beta-glucan hydrolase.

In summary, both sequence- and structure-based methods point to putative glycosyl hydrolase function with the enzymatic classification 3.2.1.x but they diverge in the suggested detailed substrates which will have to be determined experimentally.

*PF13367*: Several *Malassezia* proteins have significant HMMER hits (for example, MG7966_4204: from 106 to 292 with E-Value 1.1E-6 in *M. globosa*; MSY_1383: from 69 to 290 with E-Value 4.1E-9 in *M. sympodialis*) for PFam domain PF13367 which includes PrsW proteases. As response to antimicrobial peptides, PrsW proteases are involved in cleavage of the RsiW anti-Ϭ^W^ factor. Following two other proteolytic steps catalyzed by the RasP and ClpP proteases, the transcription factor Ϭ^W^ is released from the anti-Ϭ^W^ factor and activates transcription of genes under its control [12]. PrsW-like proteases belong to endopeptidase family M82, which is related to family M79 (**Fig 4E**) [13,14]. Recently, one protein in family M79 had a crystal structure resolved [15]. Homology modeling confirms that previously identified catalytically important residues [14,15] are conserved between these two families (**S_Fig 6**) and are located in the centre of the transmembrane bundle forming the active site (**Fig 4E**). PFam has the following annotation: “Based on predictions of the bioinformatics programme TMHMM it is likely that these residues are located on the extracytoplasmic face of PrsW placing them in a position to act as a sensor for cell envelope stress.” This is likely wrong because the related Rce1 protease shares exactly these residues and in the resolved structure they are in the catalytic core. To further corroborate this, we have used the consensus transmembrane (TM) prediction from the TMSOC method [16] that additionally allows classifying transmembrane regions into simple or complex types based on statistical Z-scores of hydrophobicity and complexity scores (**S_Table 9**, **S_Fig 14**). This is important because sequence similarity searches over simple TM regions can introduce false positive hits resulting in wrong function predictions [17]. All predicted TM regions in this case were found by TMSOC to be complex (**S_Table 9**) and likely functional (**S_Fig 14**) and the residues highlighted to be of functional importance indeed correspond to those identified as catalytic residues in the structural modeling. Consequently, an enzymatic function as protease appears credible. The M79 family includes Rce1 peptidases that typically cleave a C-terminal tripeptide from an isoprenylated protein. This is, however, not the likely detailed function of the *Malassezia* PrsW-like family because there are direct Rce1 homologues present in *Malassezia* (e.g. MGL_3383, **Fig 4E**). The detailed substrate of these new PrsW-like proteases remains to be established.

**References**

1. Eddy SR. A new generation of homology search tools based on probabilistic inference. Genome Inform Int Conf Genome Inform. 2009;23: 205–211.

2. Finn RD, Bateman A, Clements J, Coggill P, Eberhardt RY, Eddy SR, et al. Pfam: the protein families database. Nucleic Acids Res. 2014;42: D222–230.

3. Söding J, Biegert A, Lupas AN. The HHpred interactive server for protein homology detection and structure prediction. Nucleic Acids Res. 2005;33: W244–248.

4. Webb B, Sali A. Protein structure modeling with MODELLER. Methods Mol Biol Clifton NJ. 2014;1137: 1–15.

5. Sillitoe I, Lewis TE, Cuff A, Das S, Ashford P, Dawson NL, et al. CATH: comprehensive structural and functional annotations for genome sequences. Nucleic Acids Res. 2015;43: D376–381.

6. Katoh K, Standley DM. MAFFT: iterative refinement and additional methods. Methods Mol Biol Clifton NJ. 2014;1079: 131–146.

7. Mihalek I, Res I, Lichtarge O. A family of evolution-entropy hybrid methods for ranking protein residues by importance. J Mol Biol. 2004;336: 1265–1282.

8. Krieger E, Vriend G. YASARA View - molecular graphics for all devices - from smartphones to workstations. Bioinforma Oxf Engl. 2014;30: 2981–2982.

9. Tamura K, Stecher G, Peterson D, Filipski A, Kumar S. MEGA6: Molecular Evolutionary Genetics Analysis version 6.0. Mol Biol Evol. 2013;30: 2725–2729.

10. Kruppa MD, Lowman DW, Chen Y-H, Selander C, Scheynius A, Monteiro MA, et al. Identification of (1-->6)-beta-D-glucan as the major carbohydrate component of the Malassezia sympodialis cell wall. Carbohydr Res. 2009;344: 2474–2479.

11. Bairwa G, Kaur R. A novel role for a glycosylphosphatidylinositol-anchored aspartyl protease, CgYps1, in the regulation of pH homeostasis in Candida glabrata. Mol Microbiol. 2011;79: 900–913.

12. Heinrich J, Wiegert T. Regulated intramembrane proteolysis in the control of extracytoplasmic function sigma factors. Res Microbiol. 2009;160: 696–703.

13. Pei J, Grishin NV. Type II CAAX prenyl endopeptidases belong to a novel superfamily of putative membrane-bound metalloproteases. Trends Biochem Sci. 2001;26: 275–277.

14. Ellermeier CD, Losick R. Evidence for a novel protease governing regulated intramembrane proteolysis and resistance to antimicrobial peptides in Bacillus subtilis. Genes Dev. 2006;20: 1911–1922.

15. Manolaridis I, Kulkarni K, Dodd RB, Ogasawara S, Zhang Z, Bineva G, et al. Mechanism of farnesylated CAAX protein processing by the intramembrane protease Rce1. Nature. 2013;504: 301–305.

16. Wong W-C, Maurer-Stroh S, Schneider G, Eisenhaber F. Transmembrane helix: simple or complex. Nucleic Acids Res. 2012;40: W370–375.

17. Wong W-C, Maurer-Stroh S, Eisenhaber F. Not all transmembrane helices are born equal: Towards the extension of the sequence homology concept to membrane proteins. Biol Direct. 2011;6: 57.
