# Supplementary material for: Genus-Wide Comparative Genomics of Malassezia Delineates Its Phylogeny, Physiology, and Niche Adaptation on Human Skin
Source: PLoS Genet. 2015 Nov 5;11(11):e1005614. doi: 10.1371/journal.pgen.1005614 (PMC4634964; doi:10.1371/journal.pgen.1005614)
Supplement: S8 Text — (DOCX) [file pgen.1005614.s022.docx]

**S_Text 8. Gene family expansions**

*Secretory lipases (PF01764)*: Secretory lipases can be divided into two PFam families: PF03583 and PF01764. The Lipase 3 (PF01764) family includes the previously characterized *M. globosa* LIP1 gene [1]. Members of this family are also known as triglyceride lipases, and are widely distributed in prokaryotes, plants, animals (http://pfam.xfam.org/family/PF01764), and fungi (**S_Table 3**) [2]. Our analysis revealed there are eight such lipase genes in *M. globosa,* six in *M. restricta*, and seven in *M. sympodialis* (**S_Table 3**). The number of genes in other species is comparable, except in diploid *M. furfur* strains and in *M. slooffiae* (12 copies).

*Acid sphingomyelinase*: The PFam database does not have a corresponding category for acid sphingomyelinase. Therefore, we used a sequence similarity-based approach to identify these genes in the *Malassezia* proteome based on the *C. albicans* acid sphingomyelinase. We found them to be highly conserved (4-5 copies in haploids) (**S_Table 3**).

*Proteases and peptidases*: Proteases are known to be associated with virulence in pathogenic fungi [3]. The aspartyl protease family (PF00026) is among the largest domain families in the *Malassezia* genus (**S_Table 3**). The numbers of aspartyl proteases in each species vary (**S_Table 3**). For example, *M. globosa* and *M. restricta* genomes harbor more copies of this gene compared to *M. sympodialis* (**S_Table 3**). Beyond aspartyl proteases, the *Malassezia* genomes also contain a range of other proteases or peptidases (including cysteine peptidases, metallopeptidases and serine peptidases) many of which have widely varying copy numbers in different species (**S_Table 3**) and could contribute to niche specificity in *Malassezia*.

**References**

1. DeAngelis YM, Saunders CW, Johnstone KR, Reeder NL, Coleman CG, Kaczvinsky JR, et al. Isolation and Expression of a Malassezia globosa Lipase Gene, LIP1. J Invest Dermatol. 2007;127: 2138–2146.

2. Xu J, Boekhout T, DeAngelis Y, Dawson T, Saunders CW. Genomics and Pathophysiology: Dandruff as a Paradigm. In: Boekhout T, MD PM, Guého-Kellermann E, Velegraki A, editors. Malassezia and the Skin. Springer Berlin Heidelberg; 2010. pp. 253–269.

3. Monod M, Capoccia S, Léchenne B, Zaugg C, Holdom M, Jousson O. Secreted proteases from pathogenic fungi. Int J Med Microbiol. 2002;292: 405–419.
