# Supplementary material for: Genus-Wide Comparative Genomics of Malassezia Delineates Its Phylogeny, Physiology, and Niche Adaptation on Human Skin
Source: PLoS Genet. 2015 Nov 5;11(11):e1005614. doi: 10.1371/journal.pgen.1005614 (PMC4634964; doi:10.1371/journal.pgen.1005614)
Supplement: S9 Text — (DOCX) [file pgen.1005614.s023.docx]

**S_Text 9. Supplementary Methods**

***Malassezia* culture, extraction of DNA and library preparation**

After growing on modified Dixon agar for 1 week, *Malassezia* strains (see **Table 1**) were collected for genomic DNA isolation. Genomic DNA isolation was performed using the MasterPure Yeast DNA Purification Kit (Epicentre Biotechnologies) with a minor modification. Specifically, 500 μl of glass beads (425−600 nm) and 300 μl of cell lysis solution were added to the cells to assist cell wall disruption. For others steps, we followed the protocol provided by the manufacturer. We used KAPA DNA library kit (KAPA Bioscience) for library preparation. The KAPA DNA library kit uses a TruSeq Adapters (TruSeq libraries). We applied paired-end sequencing 2x100 cycles on a HiSeq2000 (Illumina) platform with a library size of 500 bp.

***Malassezia* culture, extraction of RNA and library preparation**

RNA-Seq cultures were prepared in two different media. In both cases, the inoculum was derived from a 72-hour culture of *M. globosa* 7966 in mDixon. For RNA-Seq samples from mDixon, a fresh mDixon flask was inoculated to an OD of 0.13. At the indicated time (see **Fig 4A** and **B** for details), a fraction (4 ml) of the culture was harvested by centrifugation at 4°C for ten minutes, and the supernatant was removed. The pellet was suspended in 1 ml Trizol (Life Technologies, Carlsbad, CA) and quickly frozen in a dry ice/ethanol bath. For RNA-Seq samples from minimal medium, a similar 72-hour culture from mDixon was centrifuged, washed with 0.9% sodium chloride, and suspended in 1/5^th^ the original culture volume of 0.9% sodium chloride. The cells were used to inoculate synthetic medium (15 mM ammonium sulfate, 6.6 mM monobasic potassium phosphate, 0.5 mM dibasic potassium phosphate, 1.7 mM sodium chloride, 0.7 mM calcium chloride, 2 mM magnesium chloride, 0.5 µg/ml boric acid, 0.04 µg/ml copper chloride, 0.1 µg/ml potassium iodide, 0.19 µg/ml zinc chloride, 0.05 µg/ml ferric chloride, 0.2 µg/ml calcium panthothenate, 2 µg/ml thiamine, 0.02 µg/ml biotin, 20 µg/ml inositol, 2 µg/ml pyridoxine, 2% glycerol, 1% Tween 40, 0.002 µg/ml folic acid, 0.4 µg/ml niacin, 0.2 µg/ml p-aminobenzoic acid, 0.2 µg/ml riboflavin, 0.2 µg/ml sodium molybdate, 0.4 µg/ml manganese chloride, 1 X B vitamin mix (Sigma), and 25 mM MOPS pH 6) to an OD of 0.26. At the indicated time (see **Fig 4A** and **B** for details), the culture (12 ml) was harvested and processed as described above except that 2 ml of Trizol was used.

Cell lysates were transferred into 2 ml tubes containing 0.2 ml 0.5 mm zirconia/silica beads (Biospec Products, Bartlesville, OK). Samples were homogenized on a Retsch Mixer Mill (ColeParmer, Vernon Hills, IL) for 3 minutes, incubated at room temperature, and spun at 4°C for 5 minutes at 13000 rpm. The supernatant was transferred to a new 1.5 ml tube and 200 µl of chloroform was added. Samples were vortexed, and after 3 minutes at room temperature, transferred into phaselock tubes (5prime, Gaithersburg, MD). Samples were spun at 4°C for 5 minutes at 12000 rpm, and the aqueous layer was transferred to a new 1.5 ml tube. Samples were precipitated with isopropanol and washed in fresh ice-cold 70% ethanol. The pellets were air dried and then suspended in 100 µl of RNase-free water (Life Technologies, Carlsbad, CA).

Following suspension, 35 0µl of RLT (Qiagen Inc, Valencia, CA) was added, and the sample was vortexed. Ethanol (250 µl) was added. Samples were transferred to RNeasy mini columns and purified following Qiagen’s (Qiagen Inc, Valencia, CA) method for RNA cleanup including an on-column DNase digestion. Samples were eluted in 40 µl of RNase-free water, quantified on the Nanodrop8000 (Nanodrop Products, Wilmington, DE), and quality checked on the Agilent BioAnalyzer (Agilent Technologies, Santa Clara, CA).

Starting with 1μg of total RNA we conducted an rRNA depletion step (epicenter’s Ribo-Zero Magnetic Gold Kit catalog number MRZY1306) following the manufacturer’s protocol. We checked for success of the depletion using an RNAnano chip on the Agilent Bioanalzyer. We successfully implemented the whole genome transcriptome preparation using epicenter’s ScriptSeq v2 RNA-Seq Library Preparation Kit (catalog number SSV21124) according to the manufacturer’s instructions. Library preparation entailed: end-repair, A-tailing, and ligation of adapters according to the manufacturer’s instructions. Size selection was conducted using Agencourt AMPure XP magnetic beads (Beckman Coulter, Brea, CA, USA), and fragments in the range 300-500 bp were selected. Then a quality-check of the size-selected product was run on the 2100 Bioanalyzer (DNA High Sensitivity DNA Chip). A qPCR step was preformed to ensure all material sent for sequencing contained the adaptors and indexes. We used the LightCycler 480 SYBR Green I Master mix (Roche Applied Science, Indianapolis, IN, USA) in a LightCycler® 480 II real time thermal cycler (Roche Applied Science, Indianapolis, IN, USA) according to the manufacturer’s instructions. Next generation sequencing was done using Illumina HiSeq 2000 flow cell with 2 x 76 base pair-end runs.

***Malassezia* and lipid dependence**

Two separate methods were used to extract lipids from the Bactopeptone media. A simple chloroform method was used to extract any free fatty acids, and a saponification method was applied to cleave the ester bonds on di- and triglycerides and the phospholipids, to make the potassium salt of the fatty acid, which was then converted to its acid form under low pH. Samples were analyzed by Supercritical Fluid Chromatography with a 4000 Q-trap triple quadrupole mass spectrometer (SFC/MS/MS).

A 5% solution of Bactopeptone was prepared using 0.1% formic acid in ultra-pure water. An aliquot of this sample was diluted to make a 1% solution. A blank consisted of 0.1% formic acid in ultra-pure water. To reduce contamination, all extraction vials were tripled rinsed with a 3:1 (v:v) Chloroform:methanol solution and dried before using. The chloroform method involved adding 2 mL of the sample (5%, 1%, 0% Bactopeptone) in a glass 2 dram vial, with internal standard, followed by 2 mL chloroform. Samples were vortexed for 10 seconds, and then the chloroform was transferred into a clean injection vial and dried under nitrogen.

For the saponification method, 2 mL of each solution was transferred to a clean 16x100 mm glass tube. Two ml of 20% KOH was added to each tube and heated to 60^o^C overnight. The tubes were allowed to cool; then, concentrated HCl was added drop wise until the pH fell below 2. Four mL of chloroform was added and vortexed for 30 seconds. Tubes were centrifuged at 2000 g for 15 minutes. The chloroform fraction was then transferred to an injection vial and dried.

For SFC/MS/MS analysis, the sample extracts and standards were analyzed by SFC/MS/MS using specific Multiple Reaction Monitoring (MRM) schemes for each analyte and internal standard pair with negative ion detection for the fatty acids. The concentration of each analyte in the extracts was determined by interpolation from a regression curve constructed by plotting the peak area ratio (peak area of analyte/peak area of internal standard) versus the standard concentration ranging from 20 ng to 4,000 ng.

***Malassezia* profiling from shotgun metagenomics datasets**

Accession numbers for the datasets studied in this paper are summarized below:

Skin samples [1]: all samples. The twelve individuals shown in **Fig 2** are (from left to right): HV03, HV05, HV08, HV09, HV10, HV11, HV12, HV13, HV14, HV15, SH01, SH02.

Oral samples (<http://www.hmpdacc.org/>): SRS013946, SRS014473, SRS014687, SRS015060, SRS019125, SRS017215, SRS018443, SRS023930, SRS043422, SRS046686, SRS062878, SRS013947, SRS015061, SRS019026, SRS019126, SRS063351, SRS014692, SRS015055, SRS019120, SRS104275, SRS147126, SRS077312, SRS097871, SRS143036, SRS144124, SRS148290, SRS011126, SRS017227, SRS023938, SRS024355, SRS075410, SRS013948, SRS014689, SRS019027, SRS019127, SRS065335, SRS012279, SRS022621, SRS023617, SRS050244, SRS078182 (five samples each from attached keratinized gingiva, buccal mucosa, palatine tonsil, saliva, subgingival plaque, supragingival plaque, throat, tongue dorsum, and one sample for hard palate).

Ocean samples (<http://www.microb3.eu/osd>): ERR770980, ERR770994, ERR770995, ERR771011, ERR771040, ERR771045, ERR771054.

Rhizosphere samples [2]: SRR606836, SRR606837, SRR606840, SRR606841, SRR606844, SRR606845.

Sediment samples [3]: BP101, BP101, BP120, BP120, BP139, BP139, BP143, BP143, BP155, BP155, BP186, BP186, BP278, BP278, BP444, BP444, BP463, BP463, BP501, BP501.

Soil samples [4]: mgm4477803, mgm4477804, mgm4477805, mgm4477807, mgm4477872, mgm4477873, mgm4477874, mgm4477875, mgm4477876, mgm4477877, mgm4477899, mgm4477900, mgm4477901, mgm4477902, mgm4477903, mgm4477904.

**References**

1. Kong HH, Segre JA with Oh J, Byrd AL, Deming C, Conlan S, NISC Comparative Sequencing Program. Biogeography and individuality shape function in the human skin metagenome. Nature. 2014;514: 59–64.

2. Rascovan N, Carbonetto B, Revale S, Reinert MD, Alvarez R, Godeas AM, et al. The PAMPA datasets: a metagenomic survey of microbial communities in Argentinean pampean soils. Microbiome. 2013;1: 21.

3. Mason OU, Scott NM, Gonzalez A, Robbins-Pianka A, Bælum J, Kimbrel J, et al. Metagenomics reveals sediment microbial community response to Deepwater Horizon oil spill. ISME J. 2014;8: 1464–1475.

4. Fierer N, Leff JW, Adams BJ, Nielsen UN, Bates ST, Lauber CL, et al. Cross-biome metagenomic analyses of soil microbial communities and their functional attributes. Proc Natl Acad Sci. 2012;109: 21390–21395.
