# Supplementary material for: Genus-Wide Comparative Genomics of Malassezia Delineates Its Phylogeny, Physiology, and Niche Adaptation on Human Skin
Source: PLoS Genet. 2015 Nov 5;11(11):e1005614. doi: 10.1371/journal.pgen.1005614 (PMC4634964; doi:10.1371/journal.pgen.1005614)
Supplement: S1 Table — (DOCX) [file pgen.1005614.s024.docx]

**S_Table 1. Evaluation of *de novo* assemblies from this study by comparison to corresponding reference genomes.**

|  | **Reference (MG7966)** | **Assembly (MG7966)** | **Reference (MS42132)** | **Assembly (MS42132)** |
| --- | --- | --- | --- | --- |
| Unaligned Bases | 37371 (0.42%) | 66650 | 170271 (2.21%) | 37551 |
| **Alignments** |  |  |  |  |
| 1-to-1 | 127 | 127 | 925 | 925 |
| Average Identity | 99.94 | 99.94 | 99.92 | 99.92 |
| Multiple-to-Multiple | 198 | 198 | 1020 | 1020 |
| Average Identity | 99.92 | 99.92 | 99.87 | 99.87 |
| **Feature Estimates** |  |  |  |  |
| Breakpoints | 321 | 219 | 2017 | 429 |
| Relocations | 1 | 3 | 0 | 3 |
| Translocations | 52 | 24 | 749 | 6 |
| Inversions | 0 | 0 | 2 | 2 |
| Insertions | 136 | 57 | 765 | 164 |
