# Supplementary material for: Genus-Wide Comparative Genomics of Malassezia Delineates Its Phylogeny, Physiology, and Niche Adaptation on Human Skin
Source: PLoS Genet. 2015 Nov 5;11(11):e1005614. doi: 10.1371/journal.pgen.1005614 (PMC4634964; doi:10.1371/journal.pgen.1005614)
Supplement: S2 Table — Results shown are for the M. globosa 7966 reference genome. Note that the addition of transcriptomics data (in v2, v3 and v1+v3) does not seem to improve the completeness of the identified proteome (measure by the number of PFam domains identified) but slightly improves the identification of intron-exon junctions. (DOCX) [file pgen.1005614.s025.docx]

**S_Table 2**. **Comparison of annotation quality with and without transcriptome sequencing data.** Results shown are for the *M. globosa* 7966 reference genome. Note that the addition of transcriptomics data (in v2, v3 and v1+v3) does not seem to improve the completeness of the identified proteome (measure by the number of PFam domains identified) but slightly improves the identification of intron-exon junctions.

|  |  | **Iterative annotation (v1)** | **w/ all transcripts (v2)** | **w/ selected transcripts (v3)** | **Combined annotation (v1+v3)** |
| --- | --- | --- | --- | --- | --- |
| **General Statistics** | **# of genes** | 4271 | 3779 | 4151 | 4223 |
|  | **Avg. length of genes** | 1613 | 2179 | 1789 | 1777 |
|  | **Exons per gene** | 1.92 | 2.22 | 2.02 | 2.04 |
|  | **Avg. length of exons** | 801 | 944 | 848 | 835 |
| **Proteome Completeness** | **PFam domains** | 7327 | 6667 | 7195 | 7306 |
|  | **Unique PFam domains** | 2755 | 2534 | 2699 | 2729 |
| **Intron-Exon Junctions** | **# of introns** | 3943 | 4610 | 4235 | 4371 |
|  | **Introns per gene** | 0.92 | 1.22 | 1.02 | 1.04 |
|  | **Avg. length of introns** | 79 | 68 | 75 | 75 |
|  | **Junctions** | 3940 | 4587 | 4220 | 4356 |
|  | **Supported junctions** | 2995 (76%) | 3657 (80%) | 3298 (78%) | 3417 (78%) |
