# Supplementary material for: Genus-Wide Comparative Genomics of Malassezia Delineates Its Phylogeny, Physiology, and Niche Adaptation on Human Skin
Source: PLoS Genet. 2015 Nov 5;11(11):e1005614. doi: 10.1371/journal.pgen.1005614 (PMC4634964; doi:10.1371/journal.pgen.1005614)
Supplement: S4 Table — (DOCX) [file pgen.1005614.s027.docx]

**S_Table 4**. **The distance between *PR* and *HD* loci in different *Malassezia* strains.**

| Strain | Distance (kb) |
| --- | --- |
| *M. sympodialis* reference (42132) | 141 |
| *M. globosa* reference (7966) | 168 |
| *M. globosa* 7874 | 170 |
| *M. yamatoensis* 9725 | 587 |
