# Supplementary material for: Genus-Wide Comparative Genomics of Malassezia Delineates Its Phylogeny, Physiology, and Niche Adaptation on Human Skin
Source: PLoS Genet. 2015 Nov 5;11(11):e1005614. doi: 10.1371/journal.pgen.1005614 (PMC4634964; doi:10.1371/journal.pgen.1005614)
Supplement: S5 Table — Gray area denotes “Not Determined”. The symbols +, ++ and +++ denote low but detectable, moderate, and maximal growth, while—denotes no growth. (DOCX) [file pgen.1005614.s028.docx]

**S_Table 5**. **General results of *Malassezia* lipid assimilation assay.** Gray area denotes “Not Determined”. The symbols +, ++ and +++ denote low but detectable, moderate, and maximal growth, while – denotes no growth.

| **Compound Tested** | ***M. furfur*** | ***M. sympodialis*** | ***M. globosa*** | ***M. slooffiae*** | ***M. pachydermatis**** |
| --- | --- | --- | --- | --- | --- |
| **Olive oils** |  | | | | |
| Bertolli classico Olive Oil | +++ | ++ | +++ | ++ | +/- |
| Filippo Berio Extra Virgin Olive Oil | ++ | ++ | +++ | + |  |
| Triolein (analytical standard purity) | - | - | - | - |  |
| Triolein (mixture, lab grade) | - | - | - | - | + |
| Olive Oil lab grade | ++ | ++ | ++ | - |  |
| **Polysorbitans (Tween^©^)** |  |  |  |  |  |
| Tween 20 (polyoxyethylene(20) sorbitan monolaurate) | ++ | ++ | + | ++ |  |
| Tween 40 (polyoxyethylene(20) sorbitan monopalmitate) | +++ | + | +++ | + |  |
| Tween 60 (polyoxyethylene(20) sorbitan monostearate) | +++ | ++ | +++ | + |  |
| Tween 80 (polyoxyethylene(20) sorbitan monooleate) | ++ | + | + | + |  |
| Tween 85 (polyoxyethylene(20) sorbitan monotrioleate) | +++ | + | + | + |  |
| **Animal Fats** |  | | | | |
| Lard | + |  | + |  | - |
| Artificial sebum** | ++ | + | ++ | +++ | - |
| Squalene | + |  | - |  |  |
| **Specific Lipids** |  | | | | |
| Cremphor EL (crude ricinoleic acid) | + | + | + | + |  |
| 0.1% Rincinoleic Acid (analytical standard purity) | - | - | - | - |  |
| 0.1% Stearic Acid (analytical standard purity) | - | - | - | - |  |
| 0.1% Palmitic Acid (analytical standard purity) | ++ | + | - | ++ |  |
| **Vehicles** |  | | | | |
| DMSO | - | - | - | - |  |
| PG | - | - | - | - | - |

* *M. pachydermatis* has small colonies throughout plate

** see **Methods** for details
