# Supplementary material for: Genus-Wide Comparative Genomics of Malassezia Delineates Its Phylogeny, Physiology, and Niche Adaptation on Human Skin
Source: PLoS Genet. 2015 Nov 5;11(11):e1005614. doi: 10.1371/journal.pgen.1005614 (PMC4634964; doi:10.1371/journal.pgen.1005614)
Supplement: S6 Table — Well/row letters correspond to lipid well in Fig 5A. All lipids delivered in either triolien or propylene glycol (-) controls. (-) equals no growth, 1–3 indicate low to maximal growth on a visual scale. (DOCX) [file pgen.1005614.s029.docx]

**S_Table 6. Selected lipid assimilation assay results corresponding to Fig 5A.** Well/row letters correspond to lipid well in **Fig 5A**. All lipids delivered in either triolien or propylene glycol (-) controls. (-) equals no growth, 1-3 indicate low to maximal growth on a visual scale.

| Compounds tested | *M. furfur* 7982 | *M. globosa* 7966 | *M. restricta* 7874 | Blank (no cells) |
| --- | --- | --- | --- | --- |
| A. Olive Oil | 3 | - | - | - |
| B. Propylene Glycol | - | - | - | - |
| C. Artificial Sebum* | 3 | 1 | - | - |
| D. Triolein | - | - | - | - |
| E. Wheat Germ oil | 3 | - | 1 | - |
| F. Squalene | - | - | - | - |
| G. Lard | 2 | - | - | - |
| H. Coconut oil | 2 | - | - | - |
| I. 0.1 % Rincinoleic | - | - | - | - |
| J. 0.1% Stearic | - | - | 1 | - |
| K. 0.1% Palmitic | 1 | - | - | - |
| L. Tween 20 | 2 | 1 | 1 | - |
| M. Tween 40 | 1 | 1 | - | - |
| N. Tween 60 | 2 | 2 | 1 | - |
| O. Tween 80 | 1 | - | - | - |
| P. Tween 85 | 1 | - | - | - |
| Q. 0.1% Oleic A (74%) | - | - | - | - |
| R. 0.1% Oleic A (Pure) | - | - | - | - |
| S. Linoleic | - | - | - | - |
| T. 0.1% Petroselinic | - | - | - | - |
| U. Palmitoleic | - | - | - | - |
| Blank (cells no lipid) | - | - | - | - |

* see **Methods** for details
