# Supplementary material for: Genus-Wide Comparative Genomics of Malassezia Delineates Its Phylogeny, Physiology, and Niche Adaptation on Human Skin
Source: PLoS Genet. 2015 Nov 5;11(11):e1005614. doi: 10.1371/journal.pgen.1005614 (PMC4634964; doi:10.1371/journal.pgen.1005614)
Supplement: S7 Table — The underlined assembly was found to be the optimal one (100X coverage and k-mer = 51) based on the N50 reported (the number in parentheses is the number of contigs greater than that length). (DOCX) [file pgen.1005614.s030.docx]

**S_Table 7**. **Assembly statistics for the parameters tested for isolate MR8742**. The underlined assembly was found to be the optimal one (100X coverage and k-mer = 51) based on the N50 reported (the number in parentheses is the number of contigs greater than that length).

|  |  |  | **k-mer** |  |  |
| --- | --- | --- | --- | --- | --- |
| **Coverage** | **41** | **51** | **61** | **71** | **81** |
| **621X** | 1.5kbp (655) | 31kbp (61) | 244kbp (8) | 458kbp (6) | 458kbp (6) |
| **200X** | 396kbp (8) | 462kbp (6) | 463kbp (6) | 463kbp (6) | 463kbp (6) |
| **100X** | 399kbp (7) | 463kbp (5) | 399kbp (6) | 382kbp (7) | 382kbp (7) |
| **50X** | 399kbp (7) | 399kbp (7) | 376kbp (8) | 373kbp (8) | 373kbp (8) |
