# Supplementary material for: Genus-Wide Comparative Genomics of Malassezia Delineates Its Phylogeny, Physiology, and Niche Adaptation on Human Skin
Source: PLoS Genet. 2015 Nov 5;11(11):e1005614. doi: 10.1371/journal.pgen.1005614 (PMC4634964; doi:10.1371/journal.pgen.1005614)
Supplement: S8 Table — (DOCX) [file pgen.1005614.s031.docx]

**S_Table 8. *Malassezia* genome assembly statistics.**

| **Strain** | **Total length (Mbp)** | **No. of scaffolds** | **Max length of contigs (kbp)** | **N50 (kbp)** |
| --- | --- | --- | --- | --- |
| *M. furfur* 1878 | 13.5 | 2566 | 91 | 15 |
| *M. furfur* 4172 | 14 | 2559 | 111 | 16 |
| *M. furfur* 7019 | 13.4 | 2440 | 118 | 16 |
| *M. furfur* 7710 | 14.8 | 2968 | 103 | 15 |
| *M. furfur* JPLK23 | 7.6 | 1552 | 91 | 15 |
| *M. furfur* 7982 | 7.7 | 1234 | 118 | 21 |
| *M. globosa* 7990 | 8.9 | 78 | 924 | 415 |
| *M. globosa* 7966 | 8.9 | 80 | 1575 | 724 |
| *M. globosa* 7874 | 8.9 | 95 | 1232 | 398 |
| *M. japonica* 9431 | 8.3 | 262 | 165 | 66 |
| *M. obtusa* 7876 | 7.7 | 1275 | 110 | 23 |
| *M. pachydermatis* 1879 | 8.2 | 41 | 1424 | 957 |
| *M. restricta* 7877 | 7.2 | 74 | 1036 | 403 |
| *M. restricta* 8742 | 7.3 | 57 | 1035 | 667 |
| *M. slooffiae* 7956 | 8.3 | 1429 | 97 | 16 |
| *M. sympodialis* 42132 | 7.5 | 651 | 191 | 54 |
| *M. sympodialis* 44340 | 7.5 | 603 | 283 | 60 |
| *M. sympodialis* 96806 | 7.4 | 769 | 283 | 45 |
| *M. yamatoensis* 9725 | 8.1 | 34 | 3070 | 1448 |
| *M. nana* 9557 | 7.6 | 99 | 710 | 492 |
| *M. caprae* 10434 | 7.6 | 239 | 449 | 110 |
| *M. cuniculi* 11721 | 7.5 | 83 | 1667 | 522 |
| *M. equine* 9969 | 7.7 | 123 | 900 | 372 |
| *M. dermatis* 9169 | 7.5 | 118 | 494 | 189 |
