# Supplementary material for: Genus-Wide Comparative Genomics of Malassezia Delineates Its Phylogeny, Physiology, and Niche Adaptation on Human Skin
Source: PLoS Genet. 2015 Nov 5;11(11):e1005614. doi: 10.1371/journal.pgen.1005614 (PMC4634964; doi:10.1371/journal.pgen.1005614)
Supplement: S9 Table — (DOCX) [file pgen.1005614.s032.docx]

**S_Table 9.** Detailed transmembrane region analysis for complexity and hydrophobicity as well as highlighting positions of likely functional relevance within the helices.

| **Index** | **Sequence** | **Predicted TM segment** | **Complexity** | **Hydrophobicity** | **Zscore** | **Class** |
| --- | --- | --- | --- | --- | --- | --- |
| 1 | VASVALS**P**LV**F**L**P**TLCMY**W**V**W**VCANNA | 4,30 | 2.55 | 4.48 | -2.06 | complex |
| 2 | MV**W**IYLVVSI**GG**TTIL**G**VAQLLAYLVLVSVIM**G** | 41,73 | 1.95 | 2.03 | -2.45 | complex |
| 3 | LA**G**T**W**QN**W**LLIIL**F**S**F**IMA**GGFEE**VLKYL**P**V | 102,132 | 2.49 | 0.92 | 0.10 | complex |
| 4 | TYL**DF**AVA**G**SL**G**IATV**E**CI**GF**L**H** | 149,171 | 2.56 | -3.78 | 2.71 | complex |
| 5 | **F**VTLAQ**R**LIA**G**SM**GH**ILVAVLTS**FR**AI | 185,211 | 2.55 | -0.69 | 0.48 | complex |
| 6 | SAL**W**AMA**P**SMLL**HG**SANMAV**F**IS | 221,243 | 2.79 | -0.47 | 1.82 | complex |
| 7 | MISIV**G**LY**G**NY**F**CVVCIVAILAY | 258,280 | 2.35 | 2.01 | -0.25 | complex |

**FW**: aromatic residues; **RDEH**: charged residues; **GP**: structurally important residues.
